# A newly developed eGFP-traceable recombinant rhesus lymphocryptovirus, a tool to study viral infection and replication *in vitro* and *ex vivo*, confirms gp350 as key for viral entry

**DOI:** 10.1128/jvi.00537-26

**Published:** 2026-06-11

**Authors:** Erasmus N. Kotey, Preeti Sharma, Esther Rodriguez, Jean Patrick Gonzalez-Dahua, Brenda A. Tello, Ivana G. Reidel, Gabriela M. Escalante, Yuqi Zhao, Xiwei Wu, Rodney P. Kincaid, Yan Chen, Rebecca L. Skalsky, Lorraine Z. Mutsvunguma, Javier Gordon Ogembo

**Affiliations:** 1 Department of Immuno-Oncology, Beckman Research Institute of City of Hope 2523, Duarte, California, USA; 2 Irell & Manella Graduate School of Biological Sciences of City of Hope 52483 , Duarte, California, USA; 3 Department of Integrative Genomics and Bioinformatics, Beckman Research Institute of City of Hope 2523, Duarte, California, USA; 4 Vaccine and Gene Therapy Institute, Oregon Health and Science University, Beaverton, Oregon, USA; University of Toronto, Toronto, Ontario, Canada

**Keywords:** Epstein-Barr virus, rhesus lymphocryptovirus, recombinant technology, enhanced green fluorescent protein, host range, primates, gp350, monoclonal antibodies

## Abstract

Epstein-Barr virus (EBV) is implicated in multiple epithelial and lymphoid malignancies worldwide; however, no licensed prophylactic vaccine exists, partly due to the lack of an adequate preclinical animal model to test candidate vaccines. Rhesus lymphocryptovirus (rhLCV) faithfully recapitulates EBV infection, persistence, and pathogenesis in rhesus macaques, offering an invaluable surrogate model. While recombinant EBV expressing enhanced green fluorescent protein (eGFP) enables swift identification of infected cells *in vitro* and *ex vivo*, an analogous tool for rhLCV has been lacking. Here, employing *en passant* recombination technology, we engineered a recombinant rhLCV expressing eGFP (rhLCV.eGFP) from an rhLCV bacterial artificial chromosome derived from the B-lymphoblastoid rhesus macaque cell line LCL 8664. Whole-genome sequencing confirmed sequence integrity, and functional assays demonstrated infectivity in peripheral blood mononuclear cells (PBMCs) from multiple primate species and EBV-susceptible human cell lines, and transformation competence in rhesus macaque PBMCs. To define the role of gp350/220 (gp350) in viral entry, we generated a glycoprotein gp350-deficient mutant (rhLCV.eGFPΔgp350), verified by Sanger sequencing and immunoblotting with a novel anti-gp350 monoclonal antibody (4A6). This mutant exhibited markedly reduced infectivity in rhLCV.eGFP-susceptible human B and epithelial cell lines, underscoring gp350 as a key determinant of B cell tropism, analogous to EBV gp350. Our successful engineering of rhLCV.eGFP provides a powerful platform for visualizing infection, dissecting viral entry mechanisms and cellular tropism, and advancing rhLCV as a surrogate EBV model for vaccine design, defining correlates of protection, studies of latency and reactivation, and preclinical testing of candidate antivirals and immunotherapies.

**IMPORTANCE:**

Epstein-Barr virus (EBV) infection is associated with various epithelial and lymphoid diseases. The absence of a suitable animal study model for EBV infection has hindered mechanistic studies of pathogenesis and preclinical testing of vaccines and EBV‑targeted therapies. Rhesus lymphocryptovirus (rhLCV), an EBV homolog, accurately recapitulates EBV infection and pathogenesis in rhesus macaques, offering a promising EBV surrogate model. In this study, we engineered a stable recombinant rhLCV expressing enhanced green fluorescent protein (eGFP), rhLCV.eGFP, for easy identification of infected or transformed cells *in vitro* and *ex vivo*. Further illustrating the utility of this platform, we also generated a gp350-deficient mutant rhLCV.eGFP and demonstrated markedly reduced infectivity in otherwise susceptible B and epithelial cells, confirming the EBV gp350-homologous role of rhLCV gp350 in viral entry. Thus, rhLCV.eGFP will serve as an invaluable resource for studying EBV biology and advancing the development of EBV prophylactic and therapeutic strategies using the rhLCV surrogate model.

## INTRODUCTION

Epstein-Barr virus (EBV) infection is associated with infectious mononucleosis, multiple sclerosis, and the development of several epithelial and lymphoid malignancies, accounting for 2% of all human cancers globally every year (1, 2). Following oral transmission, EBV is thought to initially infect epithelial cells in the oral mucosa; after lytic amplification, the virus is released to infect B cells, where it persists and can lead to various malignancies (3–5). A major roadblock in the development of EBV prophylactic vaccines has been the unavailability of appropriate preclinical animal models, which has limited the ability to understand how EBV initially penetrates the oral mucosa to initiate the acute primary infection and identify the *in vivo* correlates of immune protection (6). In the absence of a truly representative animal model, EBV infection mechanisms and the ability of vaccines to prevent EBV-related diseases are typically studied *in vitro, ex vivo,* and/or in suboptimal animal models (7, 8). Despite the ongoing studies of various EBV-host cell interactions, the critical combination of viral envelope glycoproteins needed for viral entry *in vivo* is still unverified. Therefore, there is a critical need for an appropriate animal model in which to study the mechanics of viral entry, investigate the establishment of infection, and provide a platform for testing novel candidate vaccines, antivirals, and immunotherapeutics (7, 9).

Currently, the most widely utilized EBV animal model is the humanized mouse, which is mainly used to study primary human B cell infection, lymphomagenesis, and T-cell-mediated responses (10–14). However, humanized mice cannot be infected orally, as they lack human epithelial cells and palatine tonsils, where the virus is thought to initially replicate, and cannot mount effective humoral immune responses. New Zealand white rabbits have also been shown to be susceptible to EBV infection, with EBV DNA detected in both peripheral blood lymphocytes and spleens of the infected animals (15–17). Similar to mice, rabbits cannot recapitulate oral infection routes, as they lack crucial aspects of human EBV pathogenesis, such as prolonged latent infection and oncogenic potential. EBV has also been shown to infect non-human primates (NHPs), such as cotton-top tamarins and common marmosets, but these models also have various limitations. Cotton-top tamarins require a high viral titer to induce lymphoma, do not effectively model persistent infection or host responses to infection, and are now unavailable for research due to their endangered status (18, 19). Common marmosets have been shown to be orally susceptible to EBV infection, but little progress has been achieved in developing this NHP as an EBV infection model due to the scarcity of animals and reagents to support characterization of infection outcomes (20–23). Together, these limitations restrict the utility of existing models in EBV research.

Rhesus lymphocryptovirus (rhLCV) is an EBV homolog that infects rhesus macaques (*Macaca mulatta*) (24). rhLCV infection of rhesus macaque recapitulates many important features of EBV infection in humans, including the host immune responses (24), and rhLCV genes can complement human EBV orthologs in nearly all viral activities (24, 25). Importantly, oral inoculation of rhesus macaque with rhLCV can result in acute infectious mononucleosis-like disease and viral shedding similar to EBV in humans, as well as establishment of persistent infection that leads to the development of virus-associated malignancies (26–29). In addition, immunosuppression of rhLCV-infected rhesus macaques, such as via iatrogenic means or co-infection with simian immunodeficiency virus, can lead to rhLCV-induced diseases, including B-cell lymphomas (28, 30). Thus, rhLCV infection in rhesus macaques offers an apt biological model for studying viral entry and assessing potential candidate vaccines, antivirals, and immunotherapeutics. However, the current lack of essential tools such as fluorescently tagged virus for *in vitro* and *ex vivo* infection and titration studies, as well as monoclonal antibodies (mAbs) targeting rhLCV glycoproteins, limits the application of rhLCV as an EBV surrogate model.

To enhance our ability to identify rhLCV-infected cells *in vitro* and *ex vivo*, we constructed an inducible recombinant lymphoblastoid cell line (LCL) 8664-derived rhLCV bacterial artificial chromosome (BAC) that expresses enhanced green fluorescent protein (eGFP) under the control of the cytomegalovirus (CMV) promoter, rhLCV.eGFP. The eGFP cassette was inserted without deleting or replacing any known functional rhLCV open reading frames (ORFs) or non-coding RNAs. Additionally, to test the utility of this model in elucidating the role of viral glycoproteins in rhLCV viral entry, we generated an rhLCV.eGFP mutant construct defective for gp350/220 (gp350), a key entry glycoprotein in the EBV infection model (31), by inserting an early stop codon in the corresponding ORF (rhLCV.eGFPΔgp350). We reconstituted each virus in the EBV-harboring cell line P3HR-1 and used the P3HR-1-derived rhLCV viruses to inoculate rhLCV-naive rhesus macaque peripheral blood mononuclear cells (PBMCs) and generate EBV-free rhLCV.eGFP- and rhLCV.eGFPΔgp350-producer LCLs. After confirming that the eGFP insertion did not affect expression of the flanking rhLCV genes, we proceeded to test the *in vitro* and *ex vivo* infectivity of recombinant viruses by flow cytometry-based eGFP detection in cells from human and different primate species. Our analysis showed that rhLCV.eGFP efficiently infected PBMCs from several Hominids and Old World monkey species, as well as multiple human cell lines, but failed to infect PBMCs from New World monkeys. In rhLCV.eGFP-susceptible cell lines, infection by the rhLCV.eGFPΔgp350 was markedly impaired, demonstrating that rhLCV gp350 is a key enhancer to infection, consistent with the established role of EBV gp350 in mediating attachment (3, 31). Together, these results show that rhLCV.eGFP enables rapid visualization of infection and detailed analysis of viral entry pathways, strengthening the rhLCV/rhesus macaque system as a surrogate platform for EBV research.

## RESULTS

### Cloning strategy and reconstitution of a puromycin-resistant recombinant eGFP-expressing rhLCV

**Fig 1:**
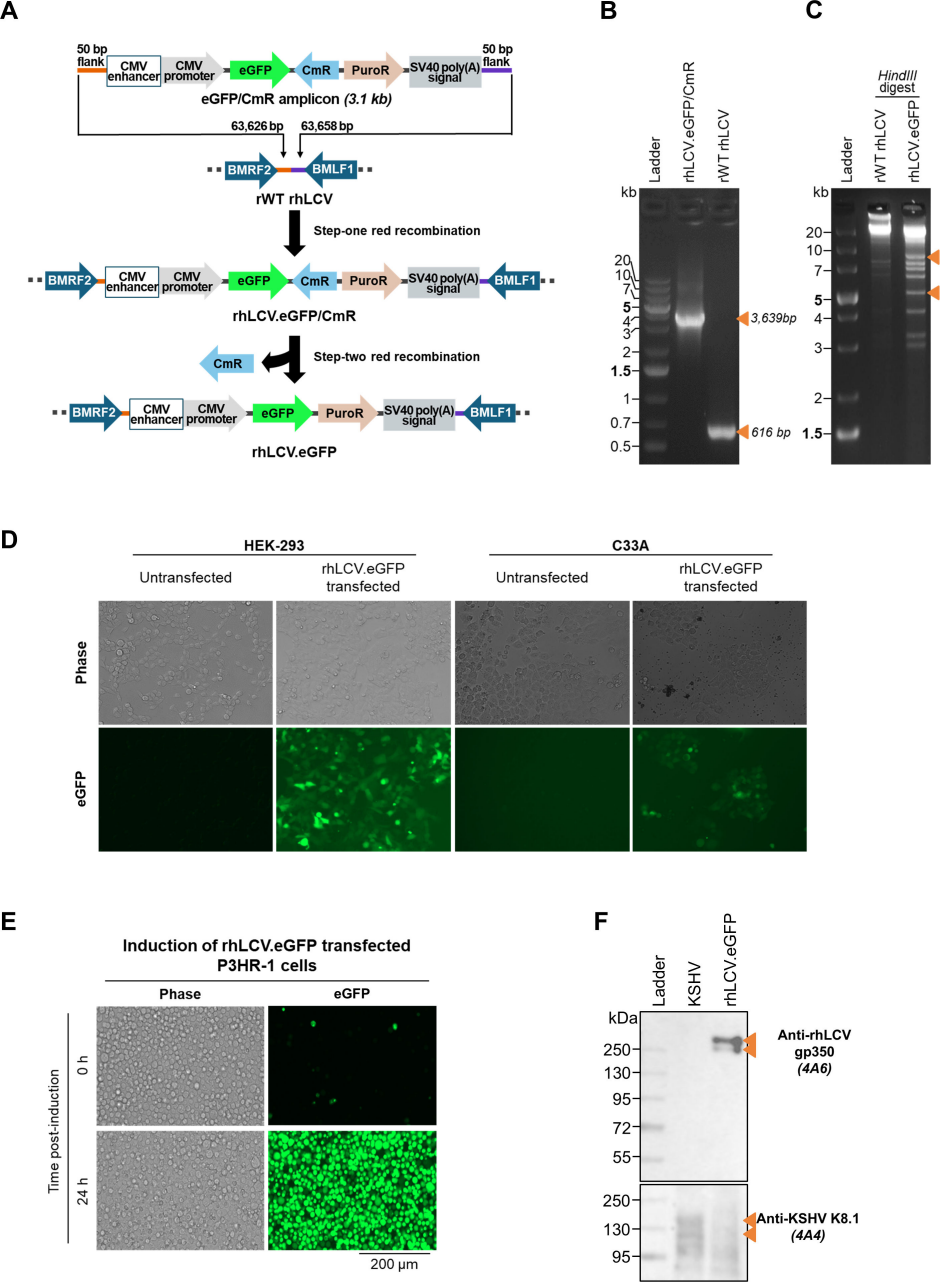
Generation of puromycin-resistant recombinant rhLCV.eGFP virus. (**A**) Schematic diagram illustrating the cloning of eGFP and PuroR genes into the rWT rhLCV BAC via *en passant* mutagenesis. During the first step, an amplicon containing the eGFP and PuroR genes, together with a CmR gene for antibiotic selection in bacteria, was integrated between the BMRF2 and BMLF1 ORFs of the genome, generating the intermediate rhLCV.eGFP/CmR. During the second step, the CmR gene was seamlessly removed to generate rhLCV.eGFP. (**B**) PCR analysis of rhLCV.eGFP/CmR DNA after the first step of *en passant* mutagenesis. Site-specific primers flanking the eGFP/Puro insertion site were used to amplify the insertion site in rhLCV.eGFP/CmR DNA, with rWT rhLCV-BAC DNA amplified as a control. Expected amplicon sizes for each sample are indicated with orange arrows. (**C**) Restriction enzyme length polymorphism (RFLP) analysis of rhLCV.eGFP DNA after the second step of *en passant* mutagenesis. rhLCV.eGFP BAC DNA was digested with Hind III enzyme and subsequently assessed by agarose gel electrophoresis, with rWT rhLCV BAC DNA digested as a control. Variations in the Hind III RFLP pattern resulting from the insertion of the eGFP and PuroR genes are indicated with orange arrows. (**D**) rhLCV.eGFP transfection of HEK-293 and C33A cells. HEK-293 and C33A cells were transfected with rhLCV.eGFP BAC DNA and maintained under puromycin selection. Shown are Phase and GFP channel micrographs of untransfected cells (left) and puromycin-selected rhLCV.eGFP-transfected cells (right) for each cell type. (**E**) Induction of rhLCV.eGFP-transfected P3HR-1 cells. P3HR-1 cells were electroporated with rhLCV-eGFP BAC DNA and maintained under puromycin selection, generating a stable rhLCV.eGFP producer P3HR-1 cell line after multiple rounds of fluorescence-activated cell sorting (FACS) enrichment for eGFP-expressing cells. Cells were subsequently induced for viral lytic replication by incubation in complete RPMI containing 12-O-tetradecanoylphorbol-13-acetate (TPA, 50 ng/mL) and NaB (3 mM) for 24 h. Shown are Phase and GFP channel micrographs of the cells before (top row) and after (bottom row) 24 h of induction. (**F**) rhLCV gp350 immunoblot analysis of P3HR1-rhLCV.eGFP. Purified P3HR1-rhLCV.eGFP and KSHV (negative control produced in iSLK rKSHV.eGFP BAC16 WT cells) were lysed and analyzed by immunoblot using mouse anti-rhLCV gp350 primary antibody 4A6, followed by horseradish peroxidase-conjugated anti-mouse secondary antibody. As a loading control for KSHV, the virus lysates were also analyzed using mouse anti-KSHV K8.1 primary antibody 4A4, followed by horseradish peroxidase-conjugated anti-mouse secondary antibody. Expected protein sizes are indicated with orange arrows.

To facilitate the study of rhLCV entry mechanisms and cellular tropism, we constructed a traceable recombinant rhLCV by introducing eGFP and puromycin resistance (PuroR) selectable marker genes into the rhLCV genome using a BAC system. We isolated the DNA of a recombinant rhLCV containing a BAC (rhLCV BAC) from BM2710 cells, which was originally engineered from a WT rhLCV genome isolated from the LCL 8664 cell line (32). The extracted DNA was then electroporated into the GS1783 *Escherichia coli* strain, which harbors the *gamma, beta,* and *exo* genes required for *en passant* mutagenesis, a two-step marker-less Red recombination system (33). Using this recombination approach, we cloned the eGFP and PuroR genes between BMRF2 and BMLF1 (Fig. 1A), without disrupting any known rhLCV ORF, generating rhLCV.eGFP BAC. During the first step of *en passant*, the eGFP and PuroR cassette was inserted into the rhLCV BAC genome, together with a chloramphenicol resistance (CmR) gene, for the easy selection of successful transformants. We confirmed successful cassette insertion in the rhLCV.eGFP/CmR BAC cloning intermediate by PCR amplification of the insertion site (Fig. 1B), using the parental rhLCV BAC as a comparator. PCR amplification using primer sets that flank the insertion site (Table S1) resulted in the expected PCR product of ~0.6 kb for rhLCV BAC DNA and a ~ 3.6 kb product for rhLCV.eGFP/CmR BAC DNA, consistent with the inserted ~3.1 kb eGFP and PuroR gene cassette containing the CmR gene. After the second step of *en passant* (CmR gene removal), we further verified successful eGFP and PuroR gene insertion in the resulting rhLCV.eGFP BAC by Hind III restriction fragment length polymorphism (RFLP) analysis (Fig. 1C). Hind III digestion resulted in different DNA banding patterns between rhLCV BAC and rhLCV.eGFP BAC DNA (Fig. 1C, orange arrows), indicative of the insertion of eGFP and PuroR genes into the rhLCV genome. To confirm sequence integrity, the rhLCV.eGFP BAC genome was sequenced by PacBio sequencing (GenBank accession number PX501811) and compared to the reference rhLCV genome GenBank ID NC_006146.1 (Table S2). We found no significant variation in the majority of ORFs. However, we found that the reference NC_006146.1 genome contained a three base-pair insertion in the gH ORF that causes three amino acid substitutions and the introduction of an additional residue, changing the motif from CGFA to LRLLP at amino acid position 614, as compared to our rhLCV.eGFP BAC sequence. When using the LRLLP sequence in mammalian protein expression constructs, gH protein expression was severely impaired when compared to constructs using the CGFA sequence, suggesting that this insertion is deleterious and possibly arose from an assembly error in the reference genome (34). Finally, to verify the functionality of the inserted eGFP cassette, we transfected human cervical cancer C33A cells and human embryonic kidney HEK-293 cells with purified rhLCV.eGFP BAC DNA, and confirmed the expression of eGFP in the corresponding transfected cells (Fig. 1D).

We next sought to reconstitute the rhLCV.eGFP virus in an inducible cell line. Thus, we evaluated cell lines previously investigated for rhLCV production as candidate producers: three EBV- and rhLCV-negative cell lines, C33A, HEK-293, and BJAB; as well as the EBV type 2-harboring human Burkitt lymphoma cell line P3HR-1 (25, 32).

We first explored the adherent cell lines C33A and HEK-293 as rhLCV.eGFP producers using a stable transfection approach. Previous work showed that lytic rhLCV replication in C33A cells depends on the overexpression of rhLCV replication and transcription activator (RTA) (BRLF1), but not the regulator of lytic replication Z transactivator (ZTA) (BZLF1) (32). In contrast, HEK-293-based EBV producer cell lines typically require ZTA for efficient virus production (32, 35–37). Thus, we tested two different approaches to achieve RTA and ZTA overexpression and therefore rhLCV.eGFP lytic replication: a transient transfection with recombinant plasmids expressing RTA or ZTA either alone or in combination, and the generation of RTA and ZTA-inducible C33A and HEK-293 stable cell lines by implementing a Tet-On inducible doxycycline system (Fig. S1A; HEK-293 data not shown). Despite these efforts, our attempts to recover any infectious virions from either cell line were futile (data not shown).

Next, we investigated whether the B cell lines BJAB or P3HR-1 cells would be suitable for rhLCV.eGFP reconstitution. We transfected both cell lines with rhLCV.eGFP BAC DNA, and upon observing eGFP expression in the transfected cells, we applied hygromycin and puromycin selection. We further enriched the eGFP-positive population through multiple rounds of fluorescence-activated cell sorting (FACS), but following selection, only eGFP-expressing P3HR-1 cells were recovered. To determine if these enriched P3HR-1 cells could produce rhLCV.eGFP virions, we induced lytic replication with 12-O-Tetradecanoylphorbol-13-acetate (TPA) and sodium butyrate (NaB) treatment, which led to an increase in eGFP expression 24 h post-induction (Fig. 1E). At 96 h post-induction, culture supernatants were ultracentrifuged, and the resulting pellet was resuspended in serum-free Opti-MEM and characterized for the presence of P3HR1-rhLCV.eGFP virions using a newly generated rhLCV gp350-specific antibody (4A6) in our laboratory as outlined below.

To generate rhLCV gp350 antibodies, we immunized BALB/c mice three times with UV-inactivated rhLCV (UV-rhLCV) produced from the LCL 8664 cell line. Mice were then boosted three additional times with either recombinant modified vaccinia Ankara (MVA) virus expressing full-length rhLCV gp350 or UV-rhLCV, followed by a final boost using purified recombinant rhLCV gp350 ectodomain (Fig. S2A). Splenocytes were harvested and fused chemically with mouse myeloma cells to generate hybridomas. Supernatants from resulting hybridoma cultures were screened by indirect ELISA for anti-gp350 reactivity, yielding 20 positive hybridoma clones (Fig. S2B). Immunoblot analysis using denatured purified rhLCV gp350 (data not shown) revealed that five hybridomas, 2A6, 3G10, 4A6, 8C11, and 9F11 (marked in color in Fig. S2B), were able to recognize linear gp350 epitopes. Antibodies purified from these clones by protein G chromatography were further evaluated by indirect ELISA, flow cytometry, and immunoblotting. All five antibodies recognized the conformational gp350 epitopes, as demonstrated by flow cytometry using BHK-21 cells infected with gp350-expressing-MVA (Fig. S2C). However, only 4A6 and 9F11 bound to linear gp350 epitopes in denatured lysates of BHK-21 cells infected with MVA expressing either untagged or His-tagged rhLCV gp350 (Fig. S2D). Given the ability of 4A6 to robustly bind to gp350 in both native and denaturing conditions, we selected it for further characterization and determined both its isotype (IgG1/kappa) and the complementarity-determining region (CDR) sequences of its variable heavy and light chains (Table S3). These sequences were cloned into recombinant antibody expression plasmids to produce and purify recombinant anti-gp350 [r4A6] antibody. ELISA confirmed that anti-gp350 [r4A6] bound immobilized rhLCV gp350 protein in a dose-dependent manner, at levels comparable to the parental hybridoma-derived antibody (Fig. S3), validating its functionality and monoclonality.

Using anti-gp350 [r4A6], we assessed the presence of gp350 in a lysate of the P3HR1-rhLCV.eGFP suspension by immunoblot analysis. A lysate from Kaposi sarcoma-associated herpesvirus (KSHV) served as a negative control for antibody specificity (Fig. 1F). Anti-gp350 [r4A6] detected gp350 in the P3HR1-rhLCV.eGFP lysate, but not in the KSHV lysate, confirming the presence of P3HR1-rhLCV.eGFP virions in the suspension. To evaluate the infectivity of this viral preparation, the P3HR1-rhLCV.eGFP suspension was used to inoculate Raji cells, a Burkitt lymphoma B cell line, using increasing inoculum volumes (Fig. S4). Fluorescence microscopy revealed eGFP-positive cells 24 h post-infection in all inoculated wells, indicating the presence of infectious virions (Fig. S4A). Flow cytometric analysis of mock- and virus-inoculated cells demonstrated a dose-dependent increase in the proportion of eGFP-expressing Raji cells with increasing inoculum volumes (Fig. S4B), thereby confirming the infectivity of P3HR1-rhLCV.eGFP.

### Generation of gp350-deficient P3HR1-rhLCV.eGFP virus

**Fig 2:**
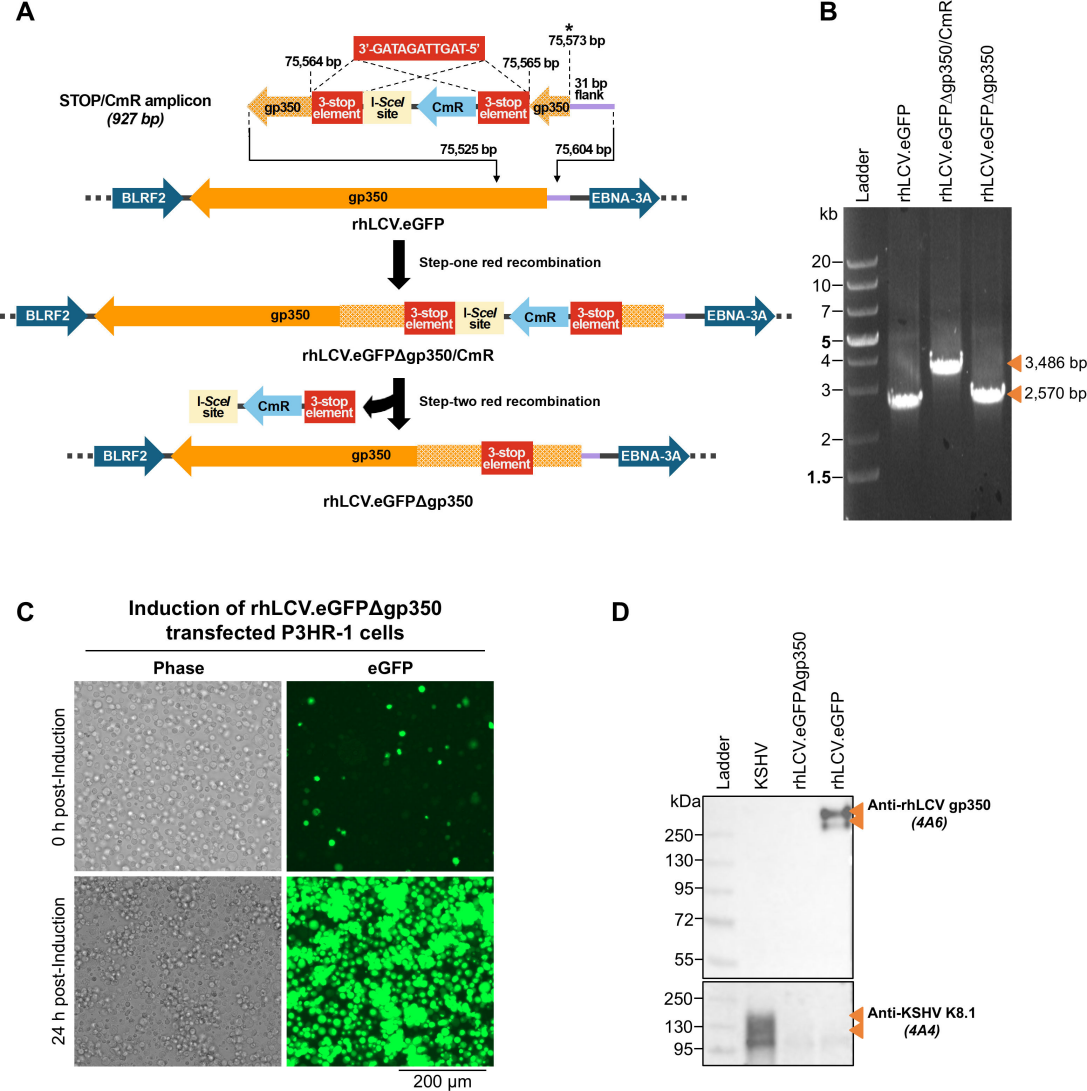
Generation of puromycin-resistant recombinant rhLCV.eGFP∆gp350 virus. (**A**) Schematic diagram illustrating the cloning of a three-stop codon element into the gp350 ORF of the rhLCV.eGFP BAC via *en passant* mutagenesis. During the first step, an amplicon containing the three-stop codon element and a CmR gene for antibiotic selection in bacteria was integrated into the coding region of gp350 between nucleotides 75,565 and 75,566, generating the intermediate rhLCV.eGFP∆gp350/CmR. During the second step, the CmR gene was seamlessly removed to generate rhLCV.eGFP∆gp350. (**B**) PCR analysis of rhLCV.eGFP∆gp350 BAC construct after the first and second *en passant* mutagenesis steps, respectively. Site-specific primers flanking the three-stop codon insertion site were used to amplify the insertion site in rhLCV.eGFP∆gp350/CmR BAC and rhLCV.eGFP∆gp350 BAC DNA, with rhLCV.eGFP BAC DNA amplified as a control. Expected amplicon sizes for rhLCV.eGFP∆gp350/CmR and rhLCV.eGFP∆gp350 are indicated with orange arrows. (**C**) Induction of rhLCV.eGFP∆gp350-transfected P3HR-1 cells. P3HR-1 cells were electroporated with rhLCV.eGFPΔgp350 BAC DNA and maintained under puromycin selection, generating a stable rhLCV.eGFP∆gp350 producer P3HR-1 cell line after multiple rounds of FACS enrichment for eGFP-expressing cells. Cells were subsequently induced for viral lytic replication by incubation in complete RPMI containing TPA (50 ng/mL) and NaB (3 mM) for 24 h. Shown are Phase and GFP channel micrographs of the cells before (top row) and after (bottom row) 24 h of induction. (**D**) rhLCV gp350 immunoblot analysis of P3HR1-rhLCV.eGFP∆gp350. Purified P3HR1-rhLCV.eGFP∆gp350, P3HR1-rhLCV.eGFP (rhLCV gp350 positive control), and KSHV (negative control produced in iSLK rKSHV.eGFP BAC16 WT cells) were lysed and analyzed by immunoblot using mouse anti-rhLCV gp350 primary antibody 4A6, followed by horseradish peroxidase-conjugated anti-mouse secondary antibody. As a loading control for KSHV, the virus lysates were also analyzed using mouse anti-KSHV K8.1 primary antibody 4A4, followed by horseradish peroxidase-conjugated anti-mouse secondary antibody. Expected protein sizes are indicated with orange arrows.

Previous studies have shown that EBV gp350 is dispensable for EBV infection of human cell lines *in vitro* and *ex vivo*, as gp350-deficient EBV can still infect B cells, albeit at a reduced efficiency (3). To determine if rhLCV gp350 is similarly dispensable, we generated a recombinant rhLCV.eGFP devoid of gp350 expression, P3HR1-rhLCV.eGFP∆gp350. Using *en passant* mutagenesis, we introduced a three-stop codon sequence (5′-TAGTTAGATAG-3′) into the early region of the gp350 ORF (BLLF1) in the rhLCV.eGFP BAC, thereby terminating protein translation in all reading frames (Fig. 2A). Successful insertion of the amplicon containing the three-stop codon element and the CmR cassette in the rhLCV.eGFP∆gp350/CmR BAC intermediate (*en passant* step one) and subsequent removal of CmR in the final rhLCV.eGFP∆gp350 BAC clone (*en passant* mutagenesis step two) were verified by PCR amplification using primers flanking the insertion site, with the parent rhLCV.eGFP BAC used as a control (Fig. 2B). As expected, insertion of the three-stop codon sequence plus CmR increased the PCR amplicon size from ~2.5 kb (parental rhLCV.eGFP BAC) to ~3.4 kb (rhLCV.eGFP∆gp350/CmR BAC), and removal of CmR restored the product to ~2.5 kb in the rhLCV.eGFP∆gp350 BAC. Finally, Sanger sequencing of rhLCV.eGFP∆gp350 BAC DNA (Fig. S5) confirmed the sequence integrity of the insertion site and in-frame insertion of the three-stop codon sequence, ensuring the truncation of gp350 during protein translation.

To reconstitute the rhLCV.eGFP∆gp350 in a producer cell line, we used the same strategy employed for P3HR1-rhLCV.eGFP. P3HR-1 cells were electroporated with rhLCV.eGFP∆gp350 BAC DNA, eGFP-positive cells were enriched by FACS, and the resulting cell population was expanded under hygromycin and puromycin selection. Following treatment of the expanded P3HR-1 cells with TPA and NaB for 24 h, we observed an increase in eGFP expression, consistent with lytic induction (Fig. 2C). After 96 h, supernatants were harvested and ultracentrifuged, and the resulting P3HR1-rhLCV.eGFP∆gp350 pellet was resuspended in serum-free Opti-MEM. We then assessed gp350 expression in this suspended virus preparation by immunoblot in parallel with P3HR1-rhLCV.eGFP (Fig. 2D). Using anti-gp350 [r4A6], gp350 was readily detected in the P3HR1-rhLCV.eGFP lysate, whereas as expected, no gp350 signal was observed in either P3HR1-rhLCV.eGFP∆gp350 lysate or the recombinant KSHV negative control virus lysate, suggesting the production of gp350-deficient rhLCV. Finally, to confirm that the mutant virus remained infectious, we inoculated Raji cells as described for P3HR1-rhLCV.eGFP. Surprisingly, fluorescence microscopy (Fig. S4A) and flow cytometry (Fig. S4B) demonstrated that P3HR1-rhLCV.eGFP∆gp350 virions efficiently infected Raji cells in a dose-dependent manner, similar to P3HR1-rhLCV.eGFP and thus contradicting prior studies of gp350-deficient EBV (3).

Because P3HR-1 cells are also EBV carriers, we asked whether the endogenous EBV was produced in parallel to the recombinant rhLCVs during induction and might be complementing P3HR1-rhLCV.eGFP∆gp350 during viral entry, which could compromise our future experiments. To answer this, we first used quantitative PCR (qPCR) to assess the presence of EBV and rhLCV in the P3HR1-rhLCV.eGFP and P3HR1-rhLCV.eGFPΔgp350 producer cell lines, their corresponding ultrapurified virus stocks (Fig. S6A), and BJAB cells infected with either virus (Fig. S6B). We designed pairs of primers targeting LMP2B of EBV and rhLCV and used published primer sets against EBV BALF5 (38) and rhLCV EBER (28) (Table S4). Both rhLCV and EBV-specific targets were consistently detected in P3HR1-rhLCV.eGFP and P3HR1-rhLCV.eGFPΔgp350 producer cell lines, their ultrapurified virus preparations, and the infected BJAB cells. These results demonstrated that the virus prepared from induced P3HR-1 cells contains a mixture of infectious EBV and recombinant rhLCV virions. Furthermore, we evaluated whether EBV gp350 could functionally complement P3HR1-rhLCV.eGFPΔgp350 entry in neutralization assays using two EBV-neutralizer mAbs, anti-EBV gp350 [72A1] (39, 40), and anti-EBV gHgL [AMMO1] (41) in Raji and BJAB cells (Fig. S6C). Consistent with prior reports of rhLCV cross-reactivity (41), anti-EBV gHgL [AMMO1] efficiently neutralized all three viruses in both cell lines. However, in contrast to previous work suggesting that anti-EBV gp350 [72A1] does not neutralize rhLCV (25), our results showed that this mAb neutralized P3HR1-rhLCV.eGFPΔgp350 in both cell lines, as well as P3HR1-rhLCV.eGFP in BJAB cells to a lower extent. These findings strongly suggest that gp350 expressed by the endogenous EBV in P3HR-1 cells can functionally complement P3HR1-rhLCV.eGFPΔgp350 and P3HR1-rhLCV.eGFP viral entry, underscoring the need to generate EBV-free producer cell lines for both recombinant viruses.

### EBV-free rhLCV.eGFP and rhLCV.eGFPΔgp350 viruses, generated from new LCLs after infection of LCV-naive rhesus macaque PBMCs, demonstrate the key role of rhLCV gp350 in Raji cell infection

**Fig 3:**
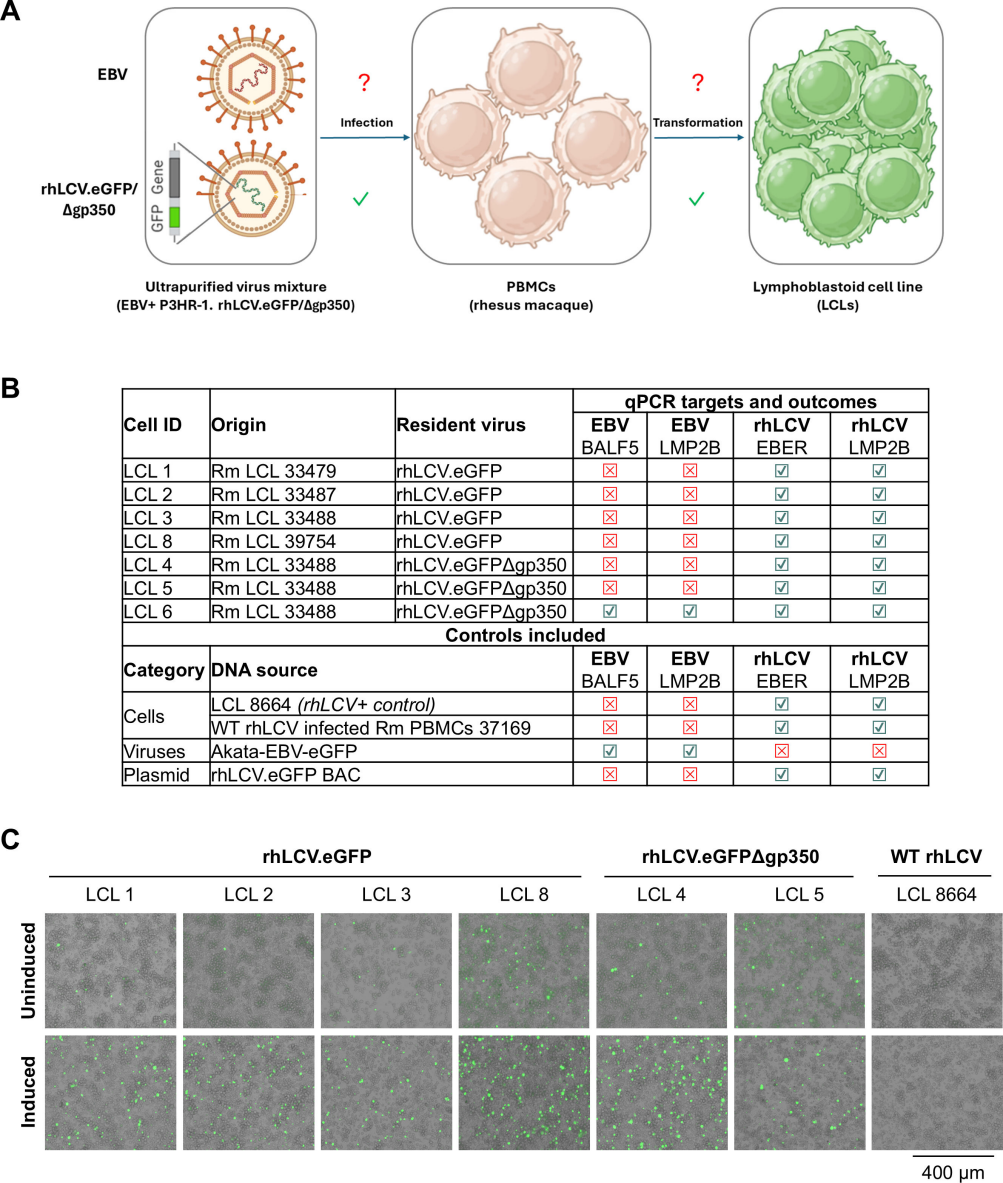
Generation of EBV-free inducible rhLCV.eGFP- and rhLCV.eGFPΔgp350-LCLs. PBMCs from various naïve rhesus macaques were inoculated with the P3HR1-rhLCV.eGFP and P3HR1-rhLCV.eGFPΔgp350 viruses and monitored until transformation into LCLs to generate rhLCV.eGFP and rhLCV.eGFPΔgp350 producer cell lines. (**A**) Schematic representation of possible EBV exclusion during infection and transformation of rhesus macaque PBMCs following P3HR1-rhLCV.eGFP/P3HR1-rhLCV.eGFPΔgp350 inoculation. (**B**) EBV genomic presence determination in resulting LCLs. DNA was extracted from all indicated cell lines, and SYBR green-based qPCR was performed to detect genomic EBV or rhLCV DNA using EBV LMP2B-, EBV BALF5-, rhLCV EBER-, and rhLCV LMP2B-specific primers (Table S4). rhLCV.eGFP BAC plasmid and DNA from LCL 8664 cells and WT rhLCV-infected rhesus macaque PBMCs were amplified as rhLCV positive controls; DNA from Akata-EBV-eGFP was amplified as an EBV-positive control. Green ticks (☑) and red crosses (⊠) represent detectable and undetectable target DNA, respectively. (**C**) Induction of EBV-free rhLCV.eGFP and rhLCV.eGFPΔgp350 LCLs. Resulting EBV-free LCLs were induced for viral lytic replication by incubation in complete RPMI containing TPA (50 ng/mL) and NaB (3 mM) for 24 h. Shown are Phase and GFP channel micrographs of the cells before (top row) and after (bottom row) 24 h of induction. LCL 8664 cells containing the WT rhLCV were used as an eGFP-negative control.

Having confirmed that endogenous EBV in P3HR-1 cells influences P3HR1-rhLCV.eGFP and P3HR1-rhLCV.eGFPΔgp350 infection, we next sought to generate EBV-free rhLCV producer cell lines. Prior studies have shown that infection and transformation of rhesus macaque PBMCs with recombinant rhLCV results in the successful establishment of LCLs as rhLCV-producer cells (25, 32), and reports indicate that EBV does not reliably transform rhesus PBMCs (34). Thus, we reasoned that infection of rhesus PBMCs with P3HR1-rhLCV.eGFP and P3HR1-rhLCV.eGFPΔgp350, followed by long-term culture until LCL outgrowth, would result in rhLCV-driven transformation and progressive loss of any EBV genomes that might initially enter these cells (Fig. 3A). To this end, PBMCs from four rhLCV-naive rhesus macaque donors were infected with P3HR1-rhLCV.eGFP or P3HR1-rhLCV.eGFPΔgp350 and cultured for 6–8 weeks, resulting in four rhLCV.eGFP LCLs and three rhLCV.eGFPΔgp350 LCLs. To determine EBV status, we performed qPCR on DNA purified from each cell line using primer pairs that target EBV BALF5, EBV LMP2B, rhLCV EBER, and rhLCV LMP2B, as described above (Fig. 3B). Controls included DNAs from LCL 8664 cells, PBMCs from a naturally rhLCV-infected macaque, purified Akata-EBV-eGFP virus, and the rhLCV.eGFP BAC DNA. As shown, all controls behaved as expected, amplifying either EBV or rhLCV genes accordingly. Importantly, all newly generated LCLs amplified only rhLCV genes, except for one rhLCV.eGFP∆gp350-derived line (LCL 6) that showed evidence of EBV co-infection and was therefore excluded from further analysis. We next assessed the inducibility of the EBV-negative LCLs by monitoring eGFP expression before and after TPA/NaB treatment (Fig. 3C). In the uninduced state, all rhLCV.eGFP and rhLCV.eGFPΔgp350 LCLs exhibited a low basal level of eGFP fluorescence, as shown by microscopy analysis (Fig. 3C, “Uninduced,” top row). Following induction, eGFP expression increased markedly in every LCL except the control (Fig. 3C, “Induced,” bottom row), consistent with activation of the lytic cycle and production of recombinant rhLCV. These findings confirm that we successfully established EBV-free inducible rhLCV.eGFP and rhLCV.eGFP∆gp350 LCLs. After evaluating small-scale virus preparations from all EBV-negative LCLs (data not shown), we selected LCL 1 and LCL 4, as optimal producer cell lines for rhLCV.eGFP and rhLCV.eGFP∆gp350, respectively, for further characterization.

First, to evaluate whether eGFP insertion affected the expression of adjacent genes, we assessed mRNA levels of the immediate upstream and downstream flanking genes, BMLF1 and BMRF2, respectively, in different induced and uninduced rhLCV producer cell lines (Fig. S7A): LCL 1 (rhLCV.eGFP), LCL 4 (rhLCV.eGFP∆gp350), LCL-rhLCV BAC (rhLCV BAC; generated in-house as a parental control), and LCL 8664 (WT-rhLCV; grandparental control). All cell lines were either mock-treated or induced with TPA and NaB and harvested at 3, 6, and 24 h for BMLF1 and BMRF2 mRNA measurements by qPCR using the primers listed in Fig. S7B. The results are expressed as RNA fold change relative to the corresponding uninduced samples using the ΔΔCT method, normalizing the target gene expression to the independent control housekeeping (HK) gene rhesus macaque Ribosomal Protein L13a (RPL13A) (42). As shown in Fig. S7A, BMLF-1 and BMRF2 transcripts increased over time after induction, with maximal upregulation at 24 h. These results are aligned with EBV reports in which both BMLF1 and BMRF2 have been described as lytic genes, the first as a modulator of the transactivation that is essential for the production of EBV infectious virions (43, 44), and the second as an envelope glycoprotein that facilitates attachment to β1 integrins on epithelial cells during infection (45, 46). The parental (LCL-rhLCV BAC) and grandparental (LCL 8664) controls displayed similar kinetic patterns, although peak expression was lower and/or delayed. While these results could suggest that the engineering process altered gene expression in different ways, these results should be interpreted with caution: traditional normalization relies on the HK gene expression staying constant; however, γ-herpesviruses can downregulate cellular mRNAs through host shut-off mechanisms during lytic phases, causing variations in HK levels (47) that can, in turn, differ across different cell lines. Nevertheless, the overall induction profiles across cell lines indicate that eGFP insertion did not impair BMLF1 or BMRF2 expression in rhLCV.eGFP or rhLCV.eGFPΔgp350, supporting preservation of flanking gene function in the engineered viruses.

**Fig 4:**
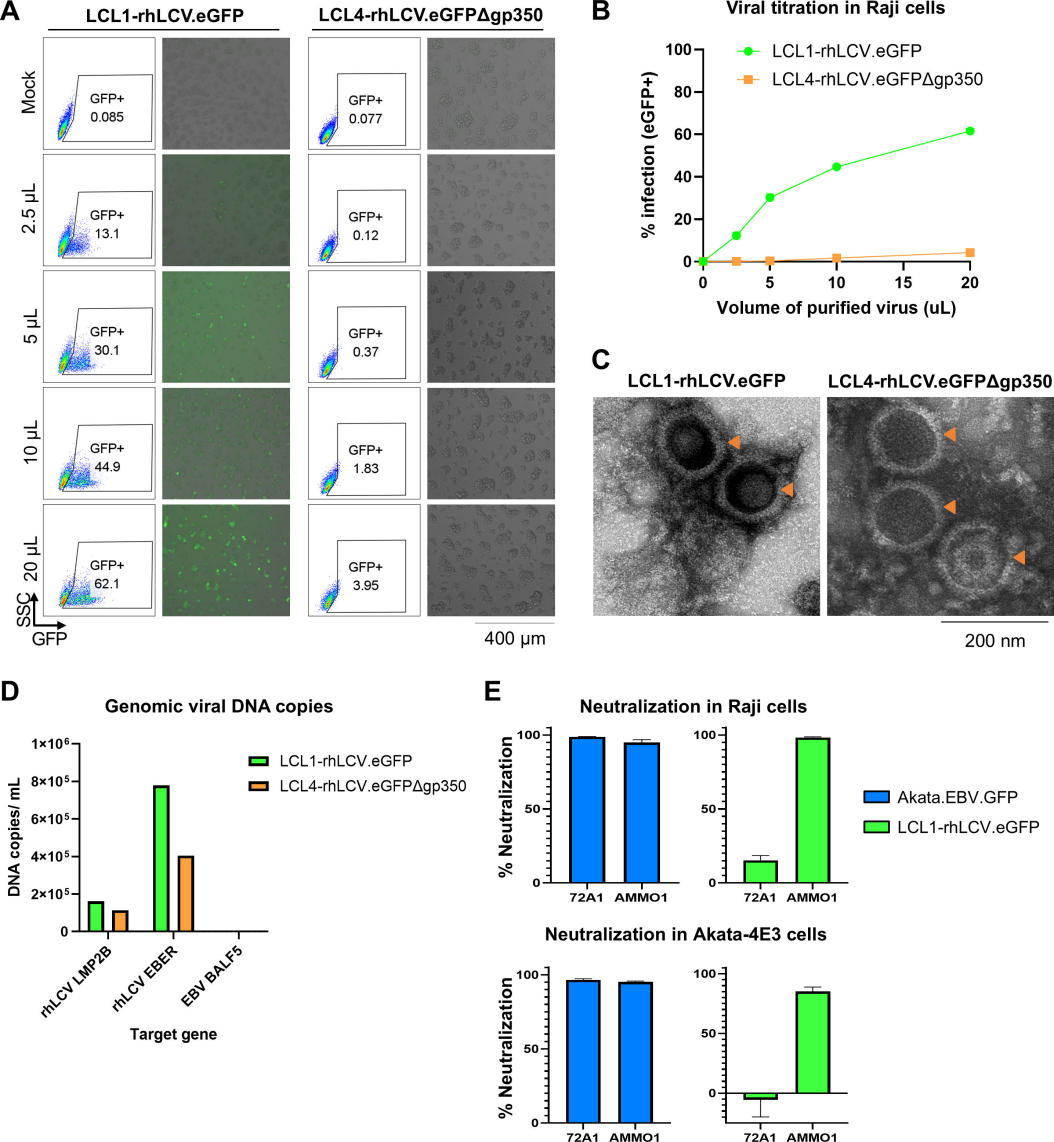
LCL1-rhLCV.eGFP and LCL4-rhLCV.eGFP∆gp350 titration in Raji cells. (**A**) Microscopy analysis of Raji cells infected with different volumes of purified LCL1-rhLCV.eGFP and LCL4-rhLCV.eGFP∆gp350 viruses. Raji cells were infected with the listed volumes of LCL1-rhLCV.eGFP or LCL4-rhLCV.eGFP∆gp350 viruses, and incubated for 24 h. Shown are representative side scatter (SSC) vs. eGFP flow cytometry plots (left) and phase-GFP-channel-merged micrographs (right) of triplicate samples for each infection volume 24 h after infection. Uninfected cells were used as a mock control. (**B**) Flow cytometry analysis of Raji cells infected with different volumes of purified LCL1-rhLCV.eGFP or LCL4-rhLCV.eGFP∆gp350 viruses. Cells from (**A**) were harvested and processed for flow cytometry analysis of eGFP expression. Shown is the mean + SEM quantification of infected (eGFP-expressing) cells at each volume for triplicates, which was used to calculate the Raji infection units (RIU)/volume for each virus as described in Methods. (**C**) Transmission electron microscopy (TEM) images of ultrapurified LCL1-rhLCV.eGFP and LCL4-rhLCV.eGFP∆gp350 viruses. Viral capsids are indicated with orange arrows. (**D**) Quantification of genomic viral DNA in the purified LCL1-rhLCV.eGFP and LCL4-rhLCV.eGFP∆gp350. Equal volumes of ultrapurified virus were treated with DNase I (0.1 U/µL) overnight, followed by DNase I deactivation, DNA extraction, and qPCR quantification of rhLCV LMP2B, rhLCV EBER, and EBV BALF5 using gBlock-based standard curves of corresponding target genes (Fig. S7). Shown is the mean of two technical replicates. (**E**) Neutralization assay to evaluate EBV complementation during LCL1-rhLCV.eGFP infection. Neutralization experiments were conducted on Raji and Akata-4E3 cell lines. Akata-EBV-eGFP (control) and LCL1-rhLCV.eGFP were pre-incubated with 25 µg of two EBV-neutralizer mAbs: anti-EBV gp350 [72A1] (non-rhLCV cross-reactive) and anti-EBV gHgL [AMMO1] (rhLCV cross-reactive). After 1 h incubation, the virus/antibody mixtures were used to inoculate Raji and Akata-4E3 cells. Viruses pre-incubated with PBS were used as infection positive controls, reaching ~15% infection in both cell lines. Shown is the average % neutralization, defined as the percentage reduction of eGFP-expressing cells in antibody-treated conditions vs. the respective positive control. All determinations were performed in triplicate.

To compare the viral production and infectivity of LCL1-rhLCV.eGFP and LCL4-rhLCV.eGFP∆gp350, we induced each producer cell line with 1 mL of induction media per 3 × 10⁶ cells and normalized the virus preparations by resuspending the ultracentrifuged pellets in 1 mL Opti-MEM per 1 L of collected supernatant. Raji cells were then inoculated with increasing volumes of LCL1-rhLCV.eGFP and LCL4-rhLCV.eGFP∆gp350 of each virus, and eGFP expression was assessed as a measure of infection (Fig. 4A and B). LCL1-rhLCV.eGFP induced a clear dose-dependent increase in eGFP-positive expression by microscopy, whereas LCL4-rhLCV.eGFPΔgp350 showed no visible infection (Fig. 4A). Flow cytometry confirmed these observations, showing that 20 μL of LCL1-rhLCV.eGFP resulted in over 60% infected Raji cells, while LCL4-rhLCV.eGFPΔgp350 only reached ~4% infection at the same input (Fig. 4B). To elucidate if these differences were due to an inefficient LCL4 viral production or an infection impediment in LCL4-rhLCV.eGFPΔgp350 associated with the gp350 deficiency, we examined the presence of virions in both purified viruses by transmission electron microscopy. Abundant viral capsids were observed in LCL1-rhLCV.eGFP and LCL4-rhLCV.eGFPΔgp350 samples, indicating efficient virion production by both producer lines, although LCL4-rhLCV.eGFPΔgp350 contained a higher proportion of empty capsids (Fig. 4C). Given this observation, we aimed to estimate the number of virions in each sample by quantifying the rhLCV DNA copies present in 1 mL of purified virus. DNase I–treated virus aliquots were subjected to DNA extraction, and rhLCV copy numbers were determined by qPCR targeting LMP2B and EBER using gBlock-based standard curves; EBV BALF5 copies were measured in parallel to assess EBV contamination (Fig. S8). qPCR against both rhLCV targets estimated that LCL1-rhLCV.eGFP contained approximately double the number of virions in comparison to LCL4-rhLCV.eGFP∆gp350 (Fig. 4D). Moreover, the absence of EBV contamination was confirmed in both LCL1-rhLCV.eGFP and LCL4-rhLCV.eGFP∆gp350. While these results confirmed the presence of virions in both recombinant viruses, our analysis is limited to the ratio between the viruses, as a reliable absolute DNA copy determination would require extended technique optimization and validation, outside of the scope of this work. Based on our analysis, a fully functional LCL4-rhLCV.eGFP∆gp350 should reach infection rates of approximately 50% of those achieved by LCL1-rhLCV.eGFP at equivalent volumes. Instead, infectivity of LCL4-rhLCV.eGFPΔgp350 in Raji cells was reduced more than 10-fold, demonstrating that gp350 loss severely compromises rhLCV infection, consistent with previous reports for EBV gp350-deficient virus (3).

Finally, to confirm that LCL1-rhLCV.eGFP infectivity was not influenced by residual EBV, we performed neutralization assays anti-EBV gp350 [72A1] and anti-EBV gHgL [AMMO1] in Raji and Akata-4E3 cells. As expected, both mAbs fully neutralized Akata-EBV-eGFP in both cell lines, whereas only anti-EBV gHgL [AMMO1] efficiently neutralized LCL1-rhLCV.eGFP, with anti-EBV gp350 [72A1] showing no measurable effect, confirming the absence of EBV-mediated complementation.

Together, these results demonstrate successful production of eGFP-expressing recombinant LCL1-rhLCV.eGFP and LCL4-rhLCV.eGFP∆gp350 viruses in WT rhLCV- and EBV-free rhesus macaque producer cell lines and show that, analogous to EBV, loss of gp350 in rhLCV results in a marked infectivity reduction in Raji cells. This recombinant platform, therefore, provides a robust model for dissecting rhLCV entry and tropism.

### LCL1-rhLCV.eGFP efficiently transforms rhLCV-naive rhesus macaque PBMCs and consistently infects the rhesus macaque-derived B cell line LCL 8664, as well as four human-derived cell lines: Raji, Akata-4E3, BJAB, and HEK-293

**Fig 5:**
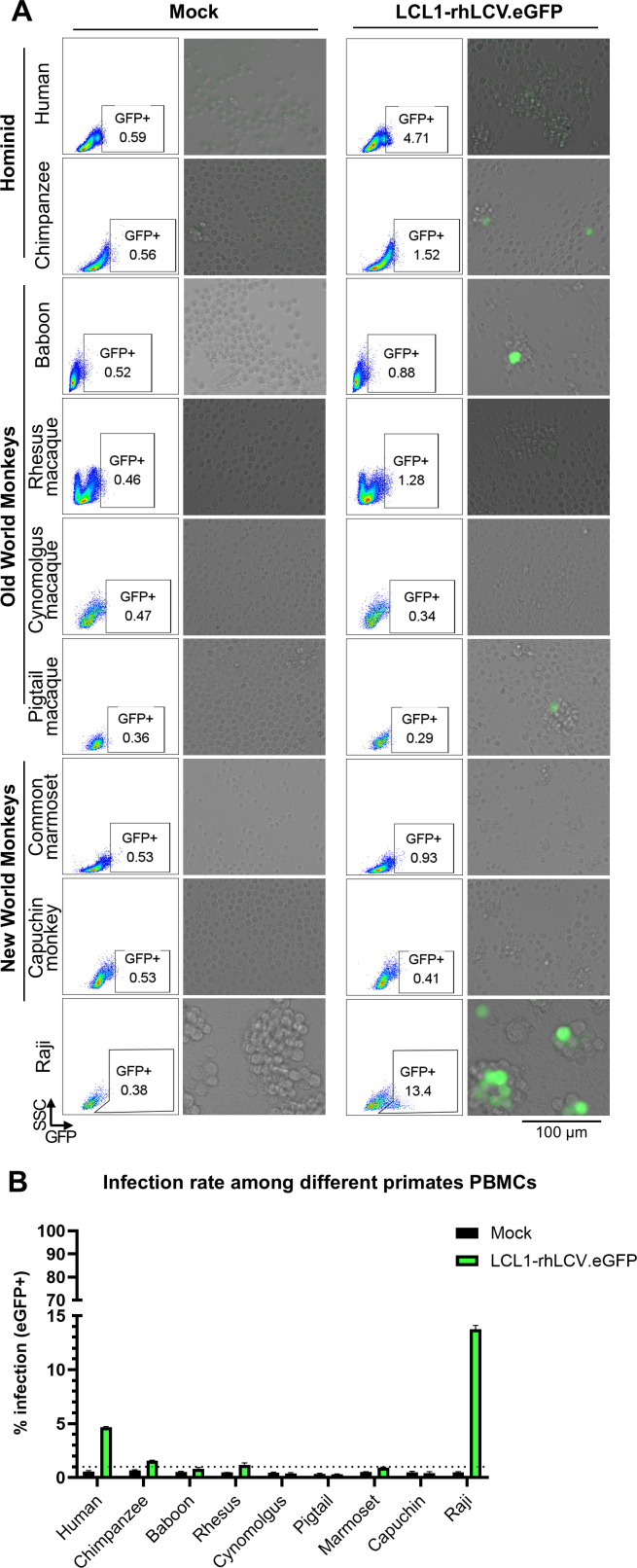
LCL1-rhLCV.eGFP infection of primate PBMCs. (**A**) LCL1-rhLCV.eGFP infection analysis of primate PBMCs. Indicated PBMCs were inoculated with 1.2 × 10^4^ RIU of purified LCL1-rhLCV.eGFP and incubated for 24 h, after which the cells were imaged, harvested, and processed by flow cytometry analysis to assess eGFP expression. Shown are representative side scatter (SSC) vs. eGFP flow cytometry plots (left) and phase-GFP-channel-merged micrographs (right) of triplicate infected samples. Raji cells were included as an LCL1-rhLCV.eGFP-susceptible cell control. (**B**) Flow cytometric quantification of eGFP-expressing infected cells. eGFP-expressing cells from panel A after infection were quantified by flow cytometry. Shown is the mean + SEM of triplicate samples for each PBMC type.

After confirming the production of EBV-free viruses, we next evaluated the *ex vivo* host range of LCL1-rhLCV.eGFP, as a model of a fully functional recombinant rhLCV. PBMCs from Hominids (human and chimpanzee), Old World monkeys (baboon, rhesus macaque, cynomolgus macaque, and pigtail macaque), and New World monkeys (capuchin monkey and common marmoset) were inoculated and examined 24 h post-infection for eGFP expression by microscopy and flow cytometry (Fig. 5A and B). LCL1-rhLCV.eGFP efficiently infected PBMCs from Hominids and among Old World monkeys, from macaque and baboon, whereas PBMCs from New World monkeys showed no detectable infection, mirroring the pattern previously observed with P3HR1–rhLCV.eGFP. While 1.28% infection in rhesus macaque PBMCs could suggest a low infectivity in the rhLCV natural target cells, this observation likely reflects that PBMCs are composed of a population of heterogeneous cells that display different levels of susceptibility to rhLCV infection, which in itself may vary across donors.

**Fig 6:**
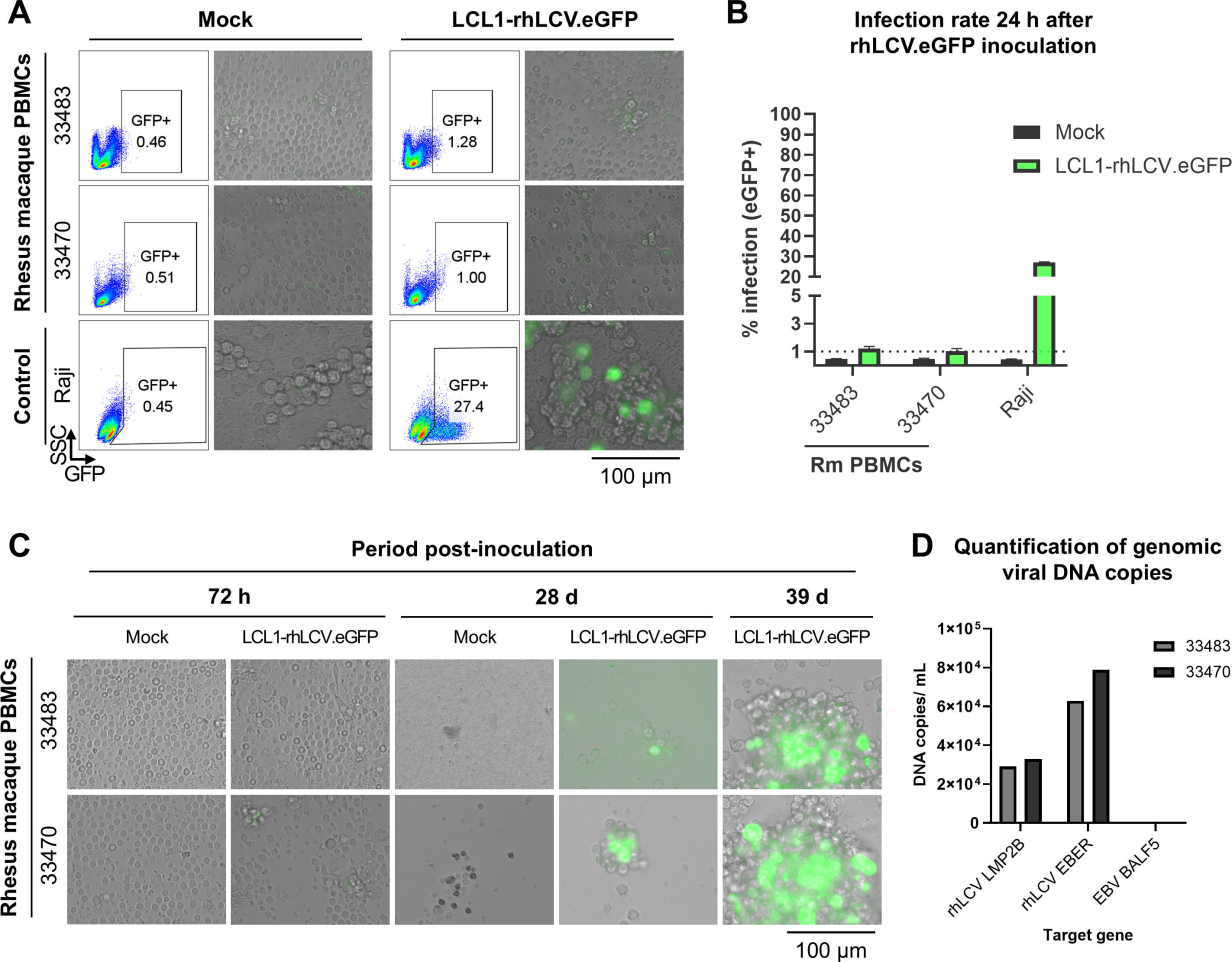
LCL1-rhLCV.eGFP transformation of rhLCV-naive rhesus macaque PBMCs. (**A**) LCL1-rhLCV.eGFP infection of rhLCV-naive rhesus macaque PBMCs. PBMCs from two animals were inoculated with 1.2 × 10^4^ RIU of purified LCL1-rhLCV.eGFP in sextuplicate and incubated for 24 h, after which the cells were imaged, three replicates were harvested, and processed by flow cytometry analysis to assess eGFP expression. Shown are representative side scatter (SSC) vs. eGFP flow cytometry plots (left) and phase-GFP-channel-merged micrographs (right) of triplicate infected samples. Raji cells were included as an LCL1-rhLCV.eGFP-susceptible cell control. Note that results from rhesus macaque 33483 were used to represent this species PBMCs infection in Fig. 5. (**B**) Flow cytometry quantification of LCL1-rhLCV.eGFP-infected rhesus macaque PBMCs. eGFP-expressing cells from panel A were quantified by flow cytometry. Shown is the mean + SEM of triplicate samples for each tested condition. (**C**) Monitoring of LCL1-rhLCV.eGFP transformation of rhesus macaque PBMCs. The remaining replicates of the LCL1-rhLCV.eGFP-infected rhesus macaque PBMCs from panel A were monitored and imaged at 72 h, 28 days, and 39 days post-inoculation. Shown are representative phase-GFP-channel-merged micrographs for each tested condition. Mock-inoculated cell controls were confirmed dead by day 28 post-inoculation; thus, imaging was not performed beyond this period for these samples. (**D**) Quantification of genomic LCL1-rhLCV.eGFP DNA in the established LCLs 2 months after initial infection. DNA was extracted from 1 × 10^6^ cells of each LCL, and qPCR quantification of rhLCV LMP 2B, rhLCV EBER, and EBV BALF5 was performed using gBlock-based standard curves of corresponding target genes (Fig. S7). Shown is the mean of two technical replicates.

Considering these limitations and that a hallmark of EBV and rhLCV is their ability to immortalize B cells from their respective hosts (34), we proceeded to evaluate whether LCL1-rhLCV.eGFP retained transforming capacity in the absence of EBV complementation. PBMCs from two rhLCV-naive rhesus macaque donors were seeded in six different wells and inoculated with LCL1-rhLCV.eGFP. Three replicates were harvested after 24 h, confirming an initial infection rate of ~1% by flow cytometry (Fig. 6A and B). The remaining replicates were maintained and continuously monitored for eGFP expression for 39 days (Fig. 6C). A distinctive eGFP signal was evident by 72 h post-inoculation, and differences between infected and mock cultures became pronounced by day 14 (data not shown), with widespread eGFP expression in infected cells; this continued expanding through day 28, at which point, transformation was considered established as mock cells were confirmed dead. By day 39, eGFP expression and cell growth were stable, allowing subculture into larger vessels. After 2 months, qPCR for rhLCV LMP2B and EBER confirmed the presence of rhLCV genomes in both resulting LCLs, and EBV BALF5 was undetectable, demonstrating that eGFP expression correlated with rhLCV-specific infection and B-cell transformation in rhesus PBMCs (Fig. 6D).

**Fig 7:**
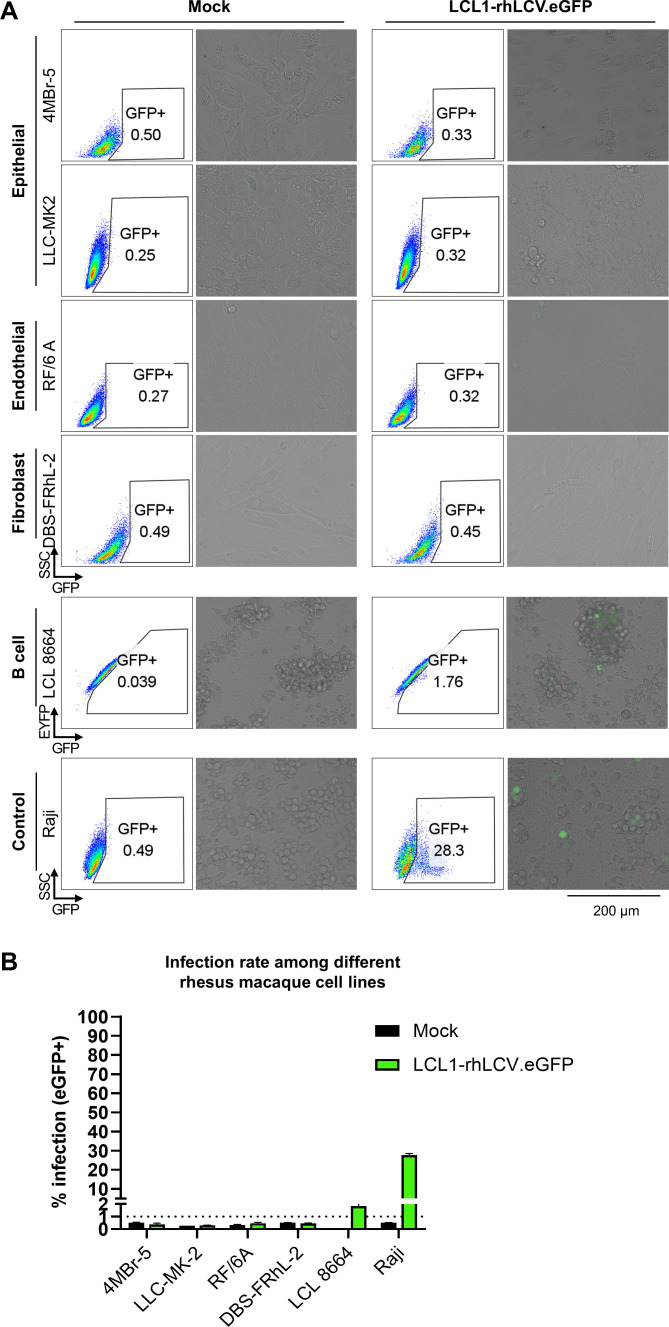
LCL1-rhLCV.eGFP infection of rhesus macaque cell lines. (**A**) LCL1-rhLCV.eGFP infection analysis of rhesus macaque cell lines. Indicated cell lines were inoculated with 1.2 × 10^4^ RIU of purified LCL1-rhLCV.eGFP virus and incubated for 24 h, after which the cells were imaged, harvested, and processed by flow cytometry to assess eGFP expression. Shown are representative side scatter (SSC) vs. eGFP flow cytometry plots (left) and phase-GFP-channel-merged micrographs (right) of triplicate infected samples. Raji cells were included as an LCL1-rhLCV.eGFP-susceptible cell control. (**B**) Flow cytometry quantification of eGFP-expressing infected cells. eGFP-expressing cells from panel A were quantified by flow cytometry. Shown is the mean + SEM of triplicate samples for each cell line.

**Fig 8:**
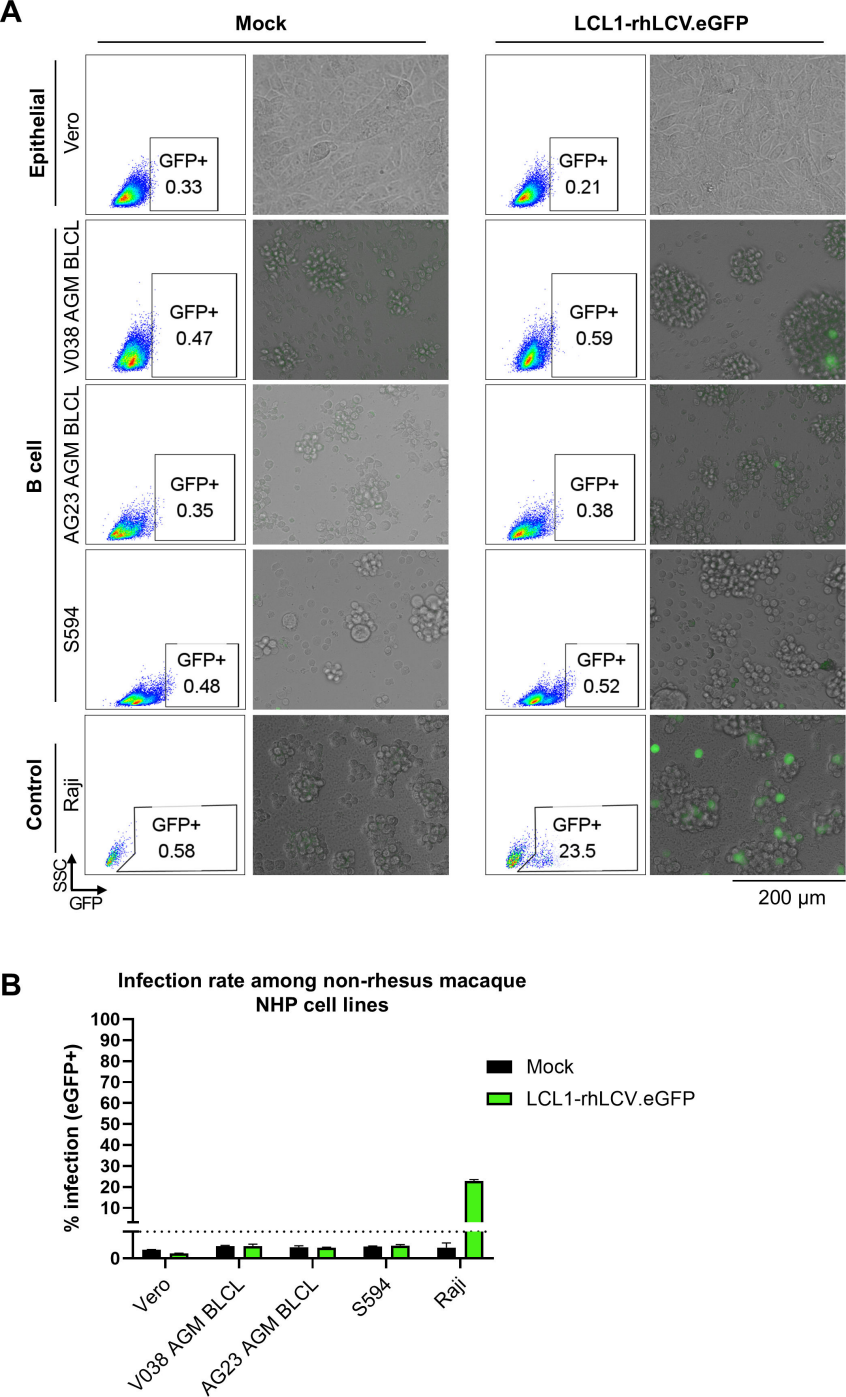
LCL1-rhLCV.eGFP infection of non-rhesus macaque NHP cell lines. (**A**) LCL1-rhLCV.eGFP infection analysis of non-rhesus macaque NHP cell lines. Indicated cell lines were inoculated with 1.2 × 10^4^ RIU of purified LCL1-rhLCV.eGFP virus, and incubated for 24 h, after which the cells were imaged, harvested, and processed by flow cytometry to assess eGFP expression. Shown are representative side scatter (SSC) vs. eGFP flow cytometry plots (left) and phase-GFP-channel-merged micrographs (right) of triplicate infected samples. Raji cells were included as an LCL1-rhLCV.eGFP-susceptible cell control. (**B**) Flow cytometry quantification of eGFP-expressing infected cells. eGFP-expressing cells from panel A were quantified by flow cytometry. Shown is the mean + SEM of triplicate samples for each cell line.

**Fig 9:**
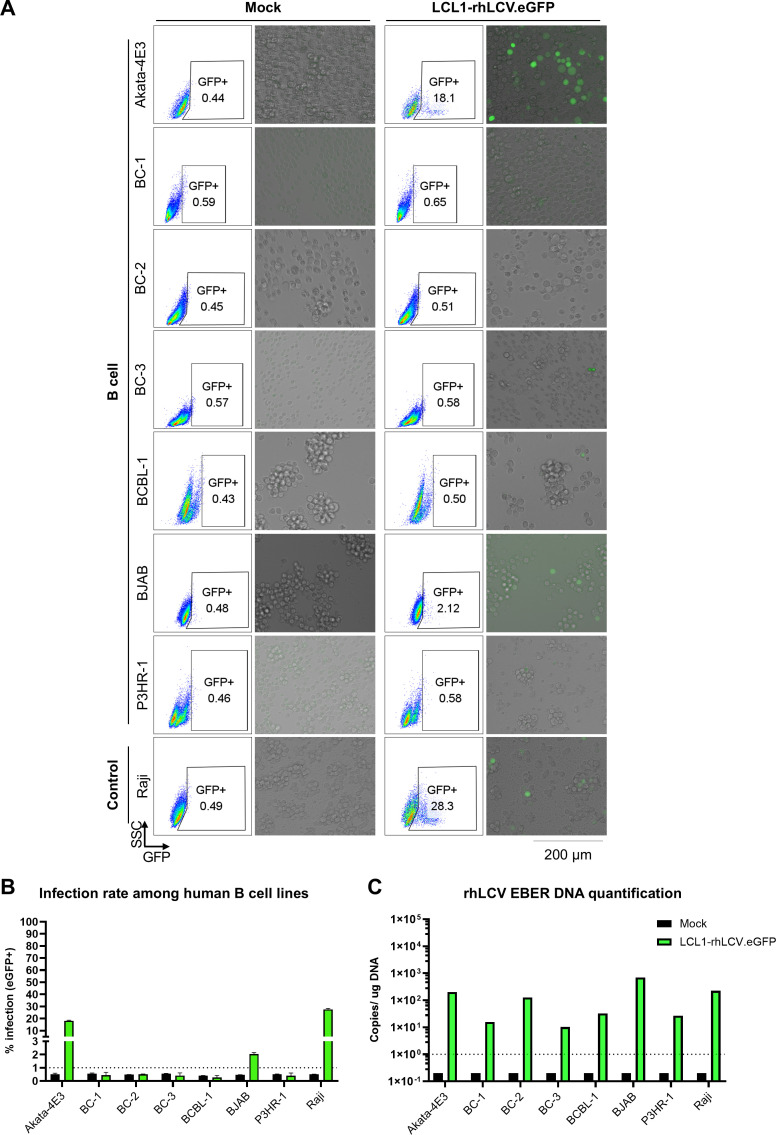
LCL1-rhLCV.eGFP infection of human B cell lines. (**A**) LCL1-rhLCV.eGFP infection analysis of human B cell lines. Indicated cell lines were inoculated with 1.2 × 10^4^ RIU of purified LCL1-rhLCV.eGFP virus and incubated for 24 h, after which the cells were imaged, harvested, and processed by flow cytometry analysis to assess eGFP expression. Shown are representative side scatter (SSC) vs. eGFP flow cytometry plots (left) and phase-GFP-channel-merged micrographs (right) of triplicate infected samples. Note that due to viability concerns with Akata 4E3, BC-2, and BJAB cells, the data displayed here were collected in three independent experiments, all including Raji cells as a positive control. Pictures and gating strategy for Raji cells were obtained in one experiment with a 27.7% average infection. This experiment was performed in parallel to the one presented in Fig. 10; thus, the same figures were used. (**B**) Flow cytometry quantification of eGFP-expressing infected cells. eGFP-expressing cells from panel A were quantified by flow cytometry. Shown is the mean + SEM of triplicate samples for each cell line. Note that due to viability concerns with Akata 4E3, BC-2, and BJAB cells, the data displayed here were collected in three independent experiments, all including Raji cells as a positive control. The results from confirmed viable conditions and the average of the three Raji determinations (mean 22.8%, 26.5%, and 27.7% infections) are included in the figure. (**C**) Quantification of genomic LCL1-rhLCV.eGFP DNA in the inoculated cell lines. Additional experimental replicates were harvested 24 h post-LCL1-rhLCV.eGFP inoculation, washed three times with PBS, and treated with DNase I (0.1 U/µL) overnight, followed by DNase I deactivation, DNA extraction, and qPCR quantification of rhLCV EBER DNA performed using a gBlock-based standard curve (Fig. S7). Shown is the mean of two technical replicates.

**Fig 10:**
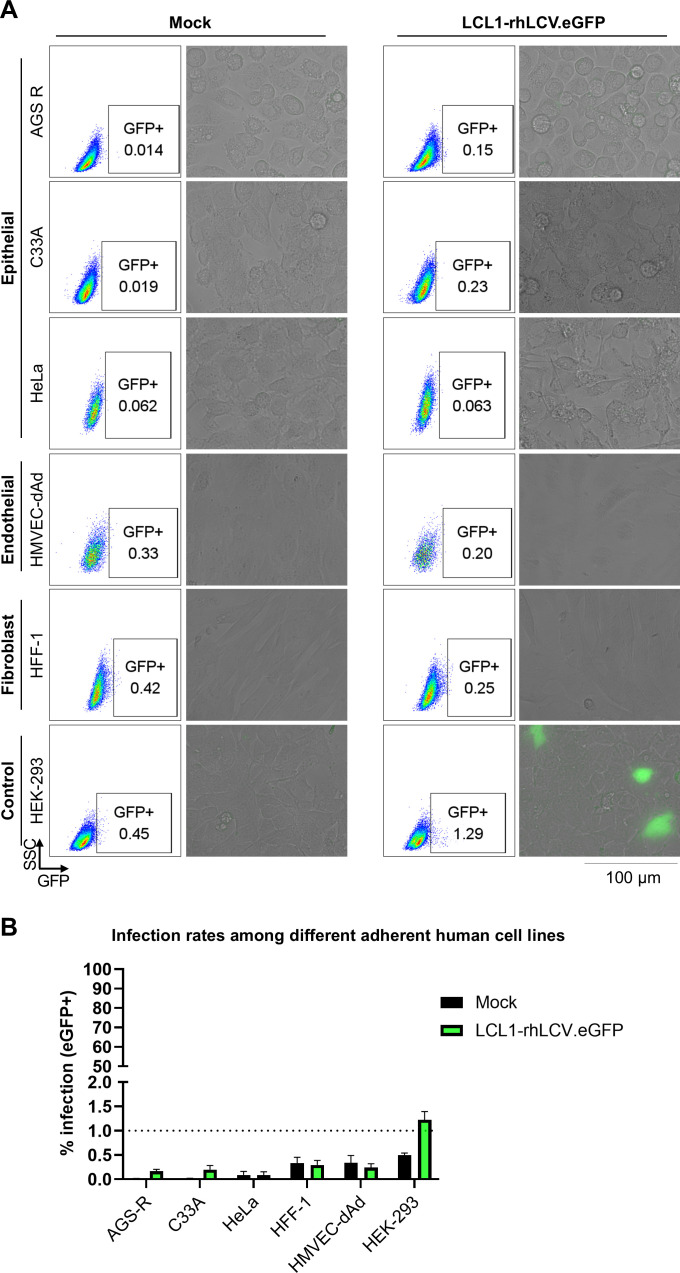
LCL1-rhLCV.eGFP infection of human epithelial, endothelial, and fibroblast cell lines. (**A**) LCL1-rhLCV.eGFP infection analysis of human epithelial, endothelial, and fibroblast cell lines. Indicated cell lines were infected with 6.0 × 10^4^ RIU of purified LCL1-rhLCV.eGFP virus and incubated for 24 h, after which the cells were imaged, harvested, and processed by flow cytometry to assess eGFP expression. Shown are representative side scatter (SSC) vs. eGFP flow cytometry plots (left) and phase-GFP-channel-merged micrographs (right) of triplicate infected samples. HEK-293 cells were included as an LCL1-rhLCV.eGFP-susceptible cell control. (**B**) Flow cytometry quantification of eGFP-expressing infected cells. eGFP-expressing cells from panel A after infection were quantified by flow cytometry. Shown is the mean + SEM of triplicate samples for each cell line.

Given that γ-herpesvirus entry depends on glycoprotein engagement with cell-type-specific receptors, and receptor expression varies among cell lines (31), we sought to define a panel of LCL1-rhLCV.eGFP-permissive cell lines for future studies. Using an inoculum that previously resulted in ~30% infection in Raji cells (Fig. 4B), we screened a set of B cell and adherent cell lines derived from rhesus macaque (Fig. 7) and other NHPs (Fig. 8). Among the five tested rhesus macaque-derived cell lines—including 4MBr-5 and LLC-MK-2 (epithelial), RF/6A (endothelial), DBS-FRhL-2 (fibroblast), and LCL 8664 (B cell)—only LCL 8664 showed signs of eGFP production after 24 h (Fig. 7A), with infection levels ~16-fold lower than those observed in the Raji control (Fig. 7B). No clear signs of infection were observed in the remaining tested NHP cell lines—including three African green monkey-derived, Vero (epithelial), V038 AGM BLCL, and AG23 AGM BLCL (B cell), and one baboon-derived, S594 (B cell)—24 h after inoculation (Fig. 8). In light of the limited susceptibility observed in the NHP cells, we decided to extend our screening to additional human cells in order to expand the panel of LCL1-rhLCV.eGFP-susceptible cell lines. We started by inoculating seven B cell lines—Akata-4E3, BC-1, BC-2, BC-3, BCBL-1, BJAB, and P3HR-1—with the same LCL1-rhLCV.eGFP inoculum volume used in the positive control Raji (Fig. 9). After 24 h, infection was microscopically clear (Fig. 9A) in Akata-4E3, BJAB, and Raji cell lines, with slight fluorescence intensity observed in BC-1 cells, as well as a low number of isolated events in the remaining cell lines when compared to the mock conditions. Despite these observations, only Akata-4E3, BJAB, and Raji presented measurable levels of eGFP by flow cytometry (Fig. 9B). To probe low-level infection in the other cell lines, we isolated DNA and quantified intracellular rhLCV EBER DNA by qPCR 24 h post-infection, following extensive washing and DNase I treatment to remove noninternalized virions and eliminate possible free rhLCV DNA in the cell suspension, respectively (Fig. 9C). Akata-4E3, BJAB, and Raji contained the highest rhLCV DNA levels, but BC-1, BC-2, BC-3, BCBL-1, and P3HR-1 also showed detectable rhLCV DNA, suggesting possible entry. While these results can suggest that all cell lines were infected with LCL1-rhLCV.eGFP, they should be interpreted with caution due to the intrinsic limitations associated with the used protocols (i.e., washes and DNase I treatment could be insufficient to remove all non-internalized rhLCV DNA). Finally, we assessed a panel of human epithelial (AGS-R, C33A, HEK-293, and HeLa), endothelial (HMVEC-dAd), and fibroblast (HFF-1) adherent cell lines for susceptibility to LCL1-rhLCV.eGFP infection (Fig. 10). Recognizing that EBV entry receptor usage is cell type-dependent (31), which could lead to variations in infection rates across susceptible cell lines despite the use of a single viral batch, we first titrated LCL1-rhLCV.eGFP on HEK-293 cells to identify a dose that yields measurable infection in adherent cells. We selected a dose equivalent to ~30% infection in Raji (Fig. 4B), estimated to be 1.2 × 10^4^ Raji infection units (RIU), and tested an increase of 5 and 10 times the starting dose (6.0 × 10^4^ and 1.2 × 10^5^ RIU, respectively). After a 24 h incubation, HEK-293 showed a dose-dependent response, with ~1%, ~3%, and ~4.5% infection at each respective dose (Fig. S9). We then used the intermediate dose (6.0 × 10^4^ RIU) to infect the adherent panel and found that only HEK-293, used as a positive control, displayed measurable eGFP at 24 h post-inoculation (Fig. 10).

In summary, these data demonstrate that LCL1-rhLCV.eGFP retained the hallmark ability of WT rhLCV to immortalize rhesus B cells and define a practical set of susceptible cell lines for future infection and intervention studies. The susceptible lines include one rhesus-derived B-cell line (LCL 8664) and four human lines known to be EBV targets (Akata-4E3, BJAB, Raji, and HEK-293), suggesting that LCL1-rhLCV.eGFP exhibits an EBV-like tropism pattern (48–50) that can be leveraged to model γ-herpesvirus entry, transformation, and antibody or antiviral effects *in vitro and ex vivo*.

### LCL4-rhLCV.eGFPΔgp350 infection is impaired in LCL1-rhLCV.eGFP-susceptible cell lines

**Fig 11:**
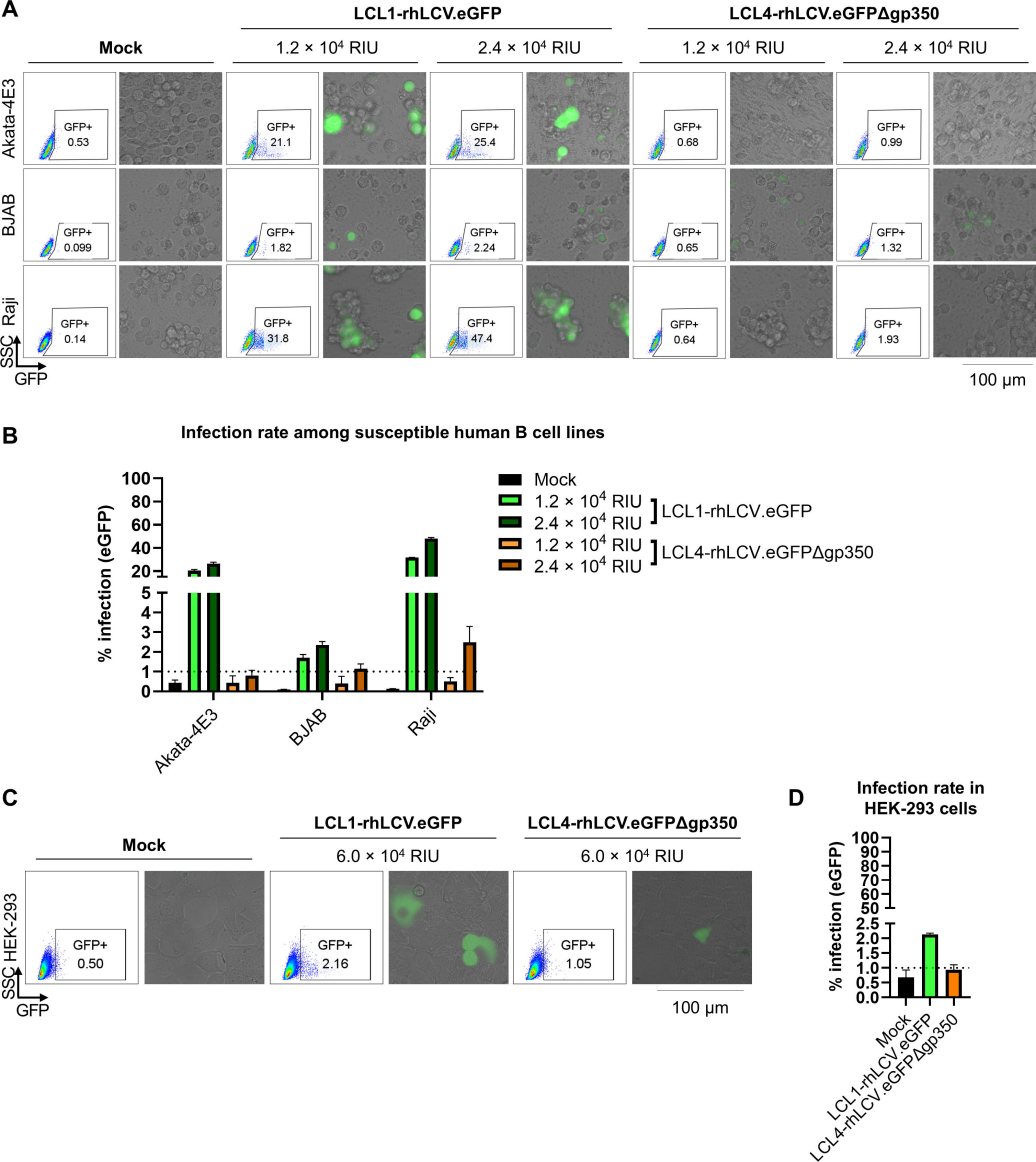
Comparison of LCL1-rhLCV.eGFP and LCL4-rhLCV.eGFPΔgp350 infectivity in susceptible cell lines. (**A**) LCL1-rhLCV.eGFP and LCL4-rhLCV.eGFP∆gp350 infection analysis in susceptible human B cell lines. Akata 4E3, BJAB, and Raji cells were inoculated with 1.2 × 10^4^ and 2.4 × 10^4^ RIU of purified LCL1-rhLCV.eGFP or LCL4-rhLCV.eGFP∆gp350 viruses, and incubated for 24 h, after which the cells were imaged, harvested, and processed by flow cytometry to assess eGFP expression. Shown are representative side scatter (SSC) vs. eGFP flow cytometry plots (left) and phase-GFP-channel-merged micrographs (right) of triplicate infected samples. (**B**) Flow cytometry quantification of eGFP-expressing infected cells. eGFP-expressing cells from panel A were quantified by flow cytometry. Shown is the mean + SEM of triplicate samples for each cell line. (**C**) LCL1-rhLCV.eGFP and LCL4-rhLCV.eGFP∆gp350 infection of HEK-293 cells. HEK-293 cells were inoculated with 6.0 × 10^4^ RIU of purified LCL1-rhLCV.eGFP or LCL4-rhLCV.eGFP∆gp350 viruses, were incubated for 24 h, after which the cells were imaged, harvested, and processed by flow cytometry to assess eGFP expression. Shown are representative side scatter (SSC) vs. eGFP flow cytometry plots (left) and phase-GFP-channel-merged micrographs (right) of triplicate infected samples. (**D**) Flow cytometry quantification of eGFP-expressing infected cells. eGFP-expressing cells from panel C were quantified by flow cytometry. Shown is the mean + SEM of triplicate samples for each cell line.

After identifying a set of LCL1-rhLCV.eGFP-susceptible cell lines, we next examined the contribution of gp350 to infection of these rhLCV-negative cell lines: Akata-4E3, BJAB, Raji, and HEK-293. We first compared LCL1-rhLCV.eGFP and LCL4-rhLCV.eGFPΔgp350 infection in the B cell lines at two viral doses (Fig. 11A and B): 1.2 × 10^4^ RIU and 2.4 × 10^4^ RIU, as estimated in LCL1-rhLCV.eGFP. Because LCL4-rhLCV.eGFPΔgp350 exhibits very low infectivity in Raji cells and thus cannot be reliably titrated (Fig. 4B), we approximated its input by using twice the LCL1-rhLCV.eGFP volume, based on qPCR data indicating that LCL4-rhLCV.eGFPΔgp350 contains roughly half as many rhLCV genomes per unit volume (Fig. 4D). At both doses, LCL1-rhLCV.eGFP achieved > 20% infection in Akata-4E3 and Raji, whereas LCL4-rhLCV.eGFPΔgp350 did not exceed ~2% infection. BJAB cells showed the same pattern with overall lower infection levels. We next assessed infection in HEK-293 cells using the previously selected LCL1-rhLCV.eGFP dose of 6.0 × 10^4^ RIU and the corresponding genome-matched volume of LCL4-rhLCV.eGFPΔgp350 (Fig. 11C and D). Under these conditions, LCL1-rhLCV.eGFP produced ~2% infection, while LCL4-rhLCV.eGFPΔgp350 reached only ~1%. Collectively, these data indicate that similar to EBV, gp350 is a major determinant of rhLCV entry into both B cells and epithelial cells, substantially enhancing infection efficiency but not being absolutely required for infection. In line with the conserved lymphocryptovirus entry machinery, other glycoproteins such as gp42, gHgL, and gB likely contribute to rhLCV attachment and fusion, providing sufficient residual function to permit low-level infection even in the absence of gp350 (29, 31).

## DISCUSSION

In virology, there has been a consolidated effort to develop methods and tools to visualize and track viruses to easily study the different stages of viral infection *in vitro, ex vivo,* and *in vivo*. These approaches have significantly enhanced the study of the life cycle of different traceable viruses such as HIV (51), Marburg virus (52), CMV (53), HSV (54), KSHV (55, 56), and EBV (57). In this study, we report for the first time the construction of a recombinant eGFP-tagged rhLCV, rhLCV.eGFP, and the generation of an inducible stable producer LCL cell line that yields high titers of rhLCV.eGFP. Moreover, we illustrate the utility of this system for generating rhLCV.eGFP mutants to evaluate the role of rhLCV glycoproteins in infection. Our data show that the incorporation of a fluorescent eGFP tag facilitates real-time monitoring of infection dynamics (*ex vivo* and *in vitro*) without requiring additional staining and enhances traditional microscopic monitoring for rhLCV-induced B cell transformation (25, 32). Such capabilities enable comprehensive studies across various cell types in a timely manner, facilitating a deeper understanding of viral behavior, including tropism, host range, and various host-pathogen interactions.

In our study, we used a BAC system and homologous recombination cloning strategies to strategically insert the eGFP tag between BMLF1 and BMRF2 without disrupting any known functional rhLCV ORFs or non-coding RNAs, thereby minimizing its impact on essential viral functions such as infectivity, replication, and egress. The fidelity of the resulting rhLCV.eGFP BAC genome was verified by PacBio sequencing, which confirmed > 90% identity in each gene across the 80 known ORFs when compared with the WT rhLCV reference sequence (LCL 8664 strain, GenBank ID NC_006146.1) (29). However, during this analysis, we identified a discrepancy within the reference sequence when compared to rhLCV.eGFP, located in the gH gene: the reference sequence contains an insertion in gH that is deleterious for recombinant gH protein expression (34), likely reflecting an early assembly error. Thus, future studies involving rhLCV gH should consider using the updated gH sequence reported here (GenBank accession number PX501811). Next, we transfected the rhLCV.eGFP BAC DNA into C33A and HEK-293 cells, which were grown under antibiotic selection, and verified the functionality of the inserted eGFP cassette. Once we confirmed the integrity and functionality of the rhLCV.eGFP BAC, we used it to generate a deletion-mutant rhLCV.eGFP devoid of gp350 expression, rhLCV.eGFP∆gp350 BAC, by inserting a sequence of three-stop codons in the early region of the gp350 ORF, to ensure premature termination of gp350 translation in all reading frames (58, 59).

Upon cloning completion, we sought to generate inducible producer cell lines for the production of both recombinant rhLCV.eGFP viruses. First, we tested the use of rhLCV.eGFP BAC-transfected C33A and HEK-293 cells, both of which were previously evaluated as recombinant rhLCV (25, 32) and EBV (60) producer cell lines due to their rhLCV/EBV-free nature. However, we were unsuccessful at recovering infectious virions from rhLCV.eGFP-transfected C33A or HEK-293 cells, even after incorporating a Tet system to overexpress the lytic replication effectors RTA and ZTA (56). As an alternative approach, we explored the transfection of an additional rhLCV/EBV-free cell line, BJAB, and the EBV-positive cell line P3HR-1, the latter of which was previously used to produce recombinant rhLCV (25). Following transfection, we enriched eGFP-positive cells by FACS and successfully established P3HR-1 as a preliminary rhLCV.eGFP producer cell line capable of producing high titers of P3HR1-rhLCV.eGFP upon TPA and NaB-mediated induction. Using the same approach, we successfully produced high titers of the mutant virus P3HR-1-rhLCV.eGFP∆gp350. Using our newly generated anti-rhLCV gp350 [r4A6] mAb, we validated the identity of the purified viruses by confirming the presence and absence of rhLCV-gp350 in P3HR1-rhLCV.eGFP and the mutant P3HR1-rhLCV.eGFPΔgp350, respectively. To the best of our knowledge, 4A6 represents the first reported rhLCV-specific gp350 mAb. Analogous gp350-specific antibodies have been critical for defining EBV gp350 biology and for developing gp350-based vaccines and immunotherapeutics (40, 61, 62). Considering anti-rhLCV gp350 [r4A6] recognizes rhLCV gp350 by immunoblot, flow cytometry, and ELISA, we expect this mAb and other clones generated in this study to become versatile tools to detect gp350 expression on virions and infected cells, facilitating future rhLCV studies.

We then confirmed the infectivity of P3HR1-rhLCV.eGFP and P3HR1-rhLCV.eGFPΔgp350 in three human B cell lines: Raji (as shown during viral titration), Akata-4E3 (data not shown), and BJAB (as shown in the neutralization assay), all highly susceptible to EBV infection (48–50). Surprisingly, unlike previous observations of reduced infectivity of gp350-knockout EBV (3), we found that P3HR1-rhLCV.eGFPΔgp350 retained the ability to infect the tested susceptible cells at levels comparable to or even higher than P3HR1-rhLCV.eGFP. Since P3HR-1 is a known EBV-2 producer cell line (63), we suspected that these observations could be linked to the co-production and co-purification of EBV with our recombinant viruses. In additional tests, we demonstrated not only the presence of EBV in both purified viruses but also that the discrepancy with the previous gp350-knockout EBV study could be due to EBV gp350 complementation during P3HR1-rhLCV.eGFPΔgp350 infection, arising from the endogenous resident EBV in the P3HR-1 producer cells. These findings underscore the need to establish new producer cell lines capable of generating uncontaminated rhLCV.eGFP viral stocks for accurate LCV infectivity studies.

To produce EBV-free rhCLV.eGFP viruses, we leveraged the fact that EBV does not transform rhesus macaque cells (64–68) and aimed to generate inducible rhesus LCLs as rhLCV.eGFP producer cell lines. Thus, we inoculated rhLCV-naive rhesus macaque PBMCs with P3HR1-rhLCV.eGFP and P3HR1-rhLCV.eGFPΔgp350, expecting that the resulting rhLCV-transformed LCLs would be free of EBV. Using this strategy, we successfully generated and selected two stable LCLs devoid of EBV genomic DNA, LCL 1 and LCL 4, for the production of EBV-free LCL1-rhLCV.eGFP and LCL4-rhLCV.eGFPΔgp350, respectively. Next, we compared the expression of BMLF1 and BMRF2, two lytic genes located upstream and downstream of the eGFP cassette, in the producer cell lines before and after induction; our results demonstrated that induction resulted in successful expression of both genes in both cell lines, suggesting that eGFP insertion did not compromise the functional integrity of the viral genome. To complete the characterization of both cell lines, we assessed the levels of LCL 1 and LCL 4 viral production and the infectivity of the resulting viruses. First, we produced ultrapurified LCL1-rhLCV.eGFP and LCL4-rhLCV.eGFPΔgp350 and showed the presence of viral capsids in each batch by TEM, confirming the efficient production of both viruses. Next, using qPCR targeting the rhLCV EBER and LMP 2B genes, we demonstrated that the LCL4-rhLCV.eGFPΔgp350 stock contained approximately half the number of DNA viral copies of those in the LCL1-rhLCV.eGFP stock. This ratio was later taken into consideration during infectivity comparison studies in different cell lines. Indeed, our preliminary viral titration in Raji cells revealed that LCL1-rhLCV.eGFP presented a quantifiable dose-dependent infection, whereas LCL4-rhLCV.eGFPΔgp350 infectivity was reduced due to its gp350 deficiency, as seen for EBV (3), compromising the accuracy of its titration. Importantly, we also showed that there were no instances of EBV protein complementation during rhCLV.eGFP infection, confirming the successful generation of EBV-free LCL1-rhLCV.eGFP and LCL4-rhLCV.eGFPΔgp350 producer lines.

Once we ensured the production of EBV-free viruses, we investigated the rhLCV host range using LCL1-rhLCV.eGFP as a model of a fully infectious recombinant rhLCV. After inoculating PBMCs from different primate species, we showed that LCL1-rhLCV.eGFP infected PBMCs from both tested Hominids (human and chimpanzee) and two of the four tested Old World monkeys (baboon and rhesus macaque), but not those from New World monkeys. Historically, rhLCV infectivity assessments relied primarily on B-cell transformation assays in the absence of a real-time trackable virus. While we demonstrated rhLCV infection in human and baboon PBMCs, prior studies reported that rhLCV failed to transform human and baboon PBMCs (65, 69), but it is unclear whether this reflected a true lack of infection or an inability to progress to immortalization. Because B cell immortalization is a hallmark of EBV and rhLCV infection in B cells from their respective hosts (34), we then inoculated PBMCs from two rhLCV-naive rhesus macaques and demonstrated that, similarly to the WT virus, LCL1-rhLCV.eGFP can efficiently transform B cells, resulting in the generation of stable eGFP-expressing LCLs. These results not only suggest that the recombinant LCL1-rhLCV.eGFP closely resemble a WT rhLCV infection but also leverage the use of this trackable system to facilitate the monitoring of *ex vivo* infection and reduce the risk of subjectivity associated with traditional B cell transformation assay readouts (70).

With this trackable LCL1-rhLCV.eGFP now available, we proceeded to test various cell lines to identify permissive models for future *in vitro* infectivity studies. Given that we had previously identified Raji cells as susceptible to rhLCV infection and used them for viral titration, we used Raji cells as a positive control in all subsequent infectivity experiments. We started by assessing the susceptibility of several rhesus macaques and other Old World monkey cell lines, including epithelial, endothelial, fibroblast, and B cell lines. Among the nine evaluated cell lines, only the LCL 8664 rhesus macaque B cell line showed measurable eGFP signals within 24 h, establishing it as a promising species-specific model for short-term assays such as the functional assessment of rhLCV-specific antibodies. Following this search for additional susceptible cell lines, we tested seven human B cell lines beyond Raji. After 24 h, we detected measurable eGFP in two of them, Akata-4E3 and BJAB, both identified as γ-herpesvirus-free cell lines that are highly susceptible to EBV infection (48–50). The five remaining human B cell lines, despite the absence of eGFP signals, presented detectable levels of intracellular rhLCV DNA; whether this represents true infection is unclear. Given that all these B cell lines harbored other herpesviruses (BC-1 and BC-2: EBV+/KSHV+; BC-3 and BCBL-1: KSHV+, P3HR-1: EBV+), our results may reflect a case of superinfection exclusion, a phenomenon in which resident viruses block entry or gene expression of a second virus (71), previously described for other herpesviruses (72, 73). Indeed, most EBV + LCLs are relatively resistant to EBV superinfection (74), with the notable exception of Raji (57), which can be superinfected with EBV as well as with rhLCV, as shown in this manuscript. Further studies beyond the scope of this work will be required to determine whether the lack of eGFP expression in the presence of rhLCV DNA truly reflects superinfection exclusion, or alternative mechanisms, limiting productive infection.

Because susceptible adherent cell lines are key to exploring rhLCV behavior in epithelial contexts, an important aspect of understanding the viral life cycle and pathogenesis (75, 76), we proceeded to explore a set of human adherent cell lines. As shown in our results, after 24 h from LCL1-rhLCV.eGFP inoculation, we could only observe a measurable eGFP signal in HEK-293 cells, which are known to be susceptible to EBV infection (77). This is not an isolated event, as the lack of true epithelial cell lines that can support EBV infection still persists. Most epithelial cell lines show < 5% EBV infectivity (e.g., AGS) with high Raji-titer virus or require CR2/CD21 expression (e.g., SVK-CR2, AGS-CR2, and HEK-293) to support higher levels of infectivity (77–80). Recently, a nasopharyngeal carcinoma cell line, HNE1, was reported to be susceptible to EBV infection (81–83), but it is not yet widely accessible. In this context, the demonstration of LCL1-rhLCV.eGFP infection in HEK-293 cells provides a practical epithelial cell platform for EBV-rhLCV comparisons and, together with the reported susceptible B cell lines, enables evaluation of rhLCV as an EBV model *in vitro*.

Once the susceptible cell lines were defined, we compared the infectivity of LCL1-rhLCV.eGFP and LCL4-rhLCV.eGFPΔgp350 in the four susceptible human cell lines. In all cases, gp350 deletion markedly reduced infectivity relative to LCL1-rhLCV.eGFP, indicating that, similar to EBV (3), gp350 plays a central role in rhLCV infection of both B cells and epithelial cells. The observation that gp350 deletion did not completely abolish rhLCV infectivity, together with the close genetic homology between rhLCV and EBV, suggests that additional entry glycoproteins such as gp42, gHgL, and gB could contribute to rhLCV entry (29), reinforcing the utility of rhLCV as an EBV infection model.

In summary, we successfully generated and characterized a trackable eGFP-tagged rhLCV, an inducible EBV-free rhLCV.eGFP producer cell lines, and the highly specific anti-rhLCV gp350 [r4A6] mAb, all of which are now available to the field. Importantly, the recombinant rhLCV.eGFP virus preserves B cell tropism and transformation competence, key features of WT EBV and rhLCV infection, which, together with the identification of permissive cell lines, demonstrate the utility of this platform as a valid EBV model. Moreover, this study highlights the robustness of our rhLCV.eGFP platform to generate rhLCV trackable mutants is an indispensable tool to easily expand the study of rhLCV viral entry, tropism, and pathogenesis.

## MATERIALS AND METHODS

### Mice

BALB/c wild-type mice were purchased from Jackson Laboratory (Bar Harbor, ME). All mice used in the experiments were housed at Beckman Research Institute of City of Hope, Duarte, CA.

### Antibodies

Anti-6×-His tag antibody was purchased from eBiosciences, San Diego, CA, for use in immunoblot. Anti-rhLCV gp350 antibodies were generated, characterized, and used to detect rhLCV gp350 in ELISA, immunoblot, and flow cytometry as described below. Horseradish peroxidase-conjugated goat-anti-mouse antibody was purchased from Millipore Sigma, Burlington, MA, for use in immunoblots and ELISA. Alexa fluor 647-conjugated anti-mouse antibody was purchased from Thermo Fisher Scientific, Waltham, MA, for use in flow cytometry.

### Cells and viruses

Human B cell lines including BC-1 (CRL-2230), BC-2 (CRL-2231), BC-3 (CRL-3615), P3HR-1 (HTB-62), Raji (CCL-86), as well as human epithelial cell lines C33A (HTB-31), HeLa (CRM-CCL-2), human epithelial kidney 293 cells (HEK-293; CRL-1573), human foreskin fibroblast 1 (HFF-1; SCRC-1041), baby hamster kidney cells 21 (BHK-21; CCL-10), P3 X 63Ag8.653 myeloma cells (CRL-1580), rhesus macaque lymphoma cells LCL 8664 (CRL-1805), rhesus macaque endothelial cells RF-6A (CRL-1780), rhesus macaque fibroblast cells DBS-FRhL-2 (CL-160), rhesus macaque epithelial cells LLC-MK2 (CCL-7) and 4MBr-5 (CCL-208), and African green monkey kidney cells Vero (CCL-81) were all obtained from American Type Culture Collection (ATCC), Manassas, VA. Primary human dermal microvascular endothelial adult cells (HMVEC-dAd; CC-2543) were obtained from Lonza Bioscience, Walkersville, MD. Gastric cancer cell line AGS-R was obtained from Dr. Lindsey Hutt-Fletcher and Dr. Rona Scott, Louisiana State University, Baton Rouge, LA. Human B cell line Akata-4E3 was obtained from Dr. Renfeng Li, Virginia Commonwealth University, VA. Human B-cell line BJAB was obtained from Dr. Joyce Fingeroth, University of Massachusetts Medical School, Worcester, MA. Human body-cavity-based lymphoma 1 (BCBL-1) cell line was obtained from Dr. Don Ganem, University of California, San Francisco, CA. The vervet monkey B cell LCL (BLCL; AG23 AGM BLCL), African green monkey BLCL (V038 AGM BLCL), and baboon LCV (S594)-infected peripheral blood lymphocytes (referred here as S594 cells) were obtained from Dr. Katherine Engelman, National Institute of Health Nonhuman Primate Reagent Resource, Boston, MA. De-identified human PBMCs were obtained from healthy adult blood donors at the Michael Amini Transfusion Medicine Center, Beckman Research Institute of City of Hope, Duarte, CA (IRB #16011). Purified non-human primate PBMCs from rhesus macaque, pigtail macaque, baboon, cynomolgus, chimpanzee, marmoset, and capuchin were obtained from the Wisconsin National Primate Research Center, Southwest National Primate Research Center, Oregon National Primate Research Center (ONPRC), or Washington National Primate Research Center. PBMCs from rhesus macaque raised in specific pathogen-free colonies were screened for prior rhLCV infection by the ONPRC and the Skalsky lab (qPCR). In this study, unless otherwise specified, all culture media were supplemented with 2 mM L-glutamine, 1% penicillin/streptomycin antibiotics, and 10% fetal bovine serum (FBS), while specific culture conditions for each cell line are detailed below. LCL 8664, V038 AGM BLCL, AG23 AGM BLCL, S594, Akata-4E3, BC-1, BC-2, BC-3, BCBL-1, BJAB, P3HR-1, Raji, and P3 X 63Ag8.653 B cells were cultured in Roswell Park Memorial Institute medium (RPMI 1640). P3HR-1 cells harboring rhLCV.eGFP BAC and gp350-deficient rhLCV.eGFPΔgp350 were maintained in complete RPMI 1640 supplemented with 100 µg/mL hygromycin B and 2 µg/mL puromycin. All PBMCs were grown in RPMI 1640 supplemented with 0.5 µg/mL cyclosporin A. Rhesus LCLs generated in this study (LCLs 1-6, LCL 8, LCL 33470, and LCL 33483) were maintained in RPMI 1640 media supplemented with 400 µg/mL hygromycin B and 2 µg/mL puromycin, while the parental rhLCV-BAC producer LCL (LCL-rhLCV BAC) was grown in RPMI 1640 media supplemented with 400 µg/mL hygromycin B. C33A, HeLa, Vero, RF-6A, and DBS-FRhL-2 cells were cultured in minimum essential medium (MEM). HEK-293, HFF-1, and BHK-21 cells were grown in Dulbecco’s modified minimal essential medium (DMEM). AGS-R cells were grown in Ham’s F-12K medium, while 4MBr-5 cells were grown in Ham’s F-12K medium supplemented with 1.5 g/L sodium bicarbonate and 30 ng/mL murine epidermal growth factor. LLC-MK2 cells were grown in media 199 supplemented with 1% horse serum instead of FBS, while HMVEC-dAd cells were cultured using an endothelial cell growth medium kit (211K-500 Cell Applications).

The iSLK cell line, encoding stable Tet-On doxycycline-inducible RTA (iSLK) under puromycin and neomycin (G418 sulfate) resistance genes, and iSLK cells harboring the rKSHV.eGFP BAC16 WT virus strain (KSHV) was obtained from Dr. Jae Jung of Lerner Research Institute, Cleveland, OH (55). The iSLK cells were maintained in DMEM supplemented with 1 µg/mL of puromycin and 250 µg/mL neomycin. iSLK rKSHV.eGFP BAC16 WT cells were cultured in iSLK cell media with the addition of 800 µg/mL of hygromycin B and used to produce KSHV infectious virions as previously described (55). Briefly, the KSHV lytic replication cycle was induced for 24 h by the addition of doxycycline (2 µg/mL) and 1.5 mM NaB, followed by 96 h of culture in DMEM + 10% FBS without any selection antibiotics. Subsequently, supernatant was collected, centrifuged in a Beckman Coulter tabletop Allegra X-14 Centrifuge at 4,000 × *g* for 30 min to remove cellular debris, and the cell-free supernatant was then filtered using 0.8 µm filters and centrifuged at 38,200 × *g* for 90 min to pellet KSHV virions. The resulting pellet was resuspended in Opti-MEM to store virus stocks.

The AGS-Akata-EBV-eGFP, a human female gastric adenocarcinoma cell line harboring EBV in which the thymidine kinase gene has been replaced with a neomycin and GFP cassette (Akata-EBV-eGFP [84]), was obtained from Dr. Lindsey Hutt-Fletcher and Dr. Rona Scott. AGS-Akata-EBV-eGFP cells were grown in DMEM/F12 supplemented with 1% L-glutamine, 2% penicillin-streptomycin, and 250 µg/mL neomycin (G418). EBV lytic replication cycle was induced for 24 h by the addition of 33 ng/mL TPA and 3 mM NaB, followed by 5–7 days of culture in growth media without G418. Cell supernatants were collected and centrifuged at 4,300 × *g* for 90 min at 4°C using a Beckman Coulter tabletop Allegra X-14 Centrifuge, filtered to remove cell debris (0.8 µm), and subjected to two rounds of sequential ultra-centrifugation in Beckman Coulter-type 19 rotors at 38,200 × *g* for 90 min at 4°C to concentrate the virus. The resulting pellets at each centrifugation were resuspended in Opti-MEM, pooled, aliquoted, and stored at −80°C.

Hybridomas expressing an anti-KSHV K8.1 antibody (4A4) were provided by Dr. Bala Chandran, University of South Florida, FL, and were cultured in RPMI 1640 supplemented with 2 mM L-glutamine, 1% penicillin/streptomycin, and 10% FBS.

Stocks of recombinant eGFP-expressing MVA virus and eGFP-expressing MVA virus also expressing native rhLCVgp350 (MVA-rhLCVgp350) or His-tagged gp350 (MVA-rhLCVgp350.His) were produced in-house in BHK-21 cells following previously established protocols (85–87).

GS1783 *Escherichia coli* cells and chloramphenicol-resistant BM2710 *Escherichia coli* cells harboring recombinant kanamycin-resistant rWT rhLCV BAC DNA originating from LCL 8664 (32) were obtained from Dr. Don Diamond of the Beckman Research Institute of City of Hope and Dr. Frederick Wang of Brigham and Women’s Hospital, Harvard Medical School, respectively, and were cultured in standard LB agar and broth.

### Construction of the puromycin-selectable rhLCV.eGFP BAC and gp350-deficient rhLCV.eGFPΔgp350 BAC constructs

To generate the recombinant puromycin-resistant rhLCV.eGFP BAC, we isolated rWT rhLCV BAC DNA from BM2710 cells using the PureLink Genomic DNA kit (Thermo Fisher Scientific); 2 µg of purified rWT rhLCV BAC DNA was electroporated into GS1783 *E. coli* strain using a 0.1-cm cuvette at the following parameters: 1.8 kV, 200 Ω, and 25 μF. Expression cassettes for the eGFP gene and puromycin resistance genes were introduced into the rWT rhLCV genome in transformed GS1783 via the two-step scarless Red recombination *en passant* mutagenesis techniques as previously described (33). Briefly, we first modified the pEGFP-puro plasmid obtained from Michael McVoy (Addgene plasmid #45561; http://n2t.net/addgene:45561; RRID: Addgene_45561) by inserting a chloramphenicol resistance gene preceded by an I-SceI restriction enzyme site and flanked by short homologous sequences between the eGFP and puromycin genes. We then PCR-amplified the eGFP, CmR, and PuroR genes from the modified pEGFP-puro plasmid using the primer pair listed in Table S1; 2 µL of the eGFP-CmR-PuroR PCR product was electroporated into competent GS1783 cells harboring the rWT rhLCV BAC genome as described above. Colony PCR using specific primers (Table S1) was used to identify and select positive clones after culture in chloramphenicol-selection medium. For scarless removal of the CmR gene from the inserted sequences, we initiated I-SceI-mediated second Red-recombination reaction via arabinose treatment as previously described (33). Colony PCR using specific primers (Table S1) was used again to identify clones with successful CmR gene removal. The insertion of the eGFP and PuroR genes, as well as the removal of CmR, was verified by clonal PCR and Sanger sequencing using specific primer sets listed in Table S1. Next, restriction fragment length polymorphism analysis was conducted on whole-genome DNA from rWT rhLCV and rhLCV.eGFP isolated from GS1783 *E. coli* using *Hind* III restriction digest and analyzed via agarose gel electrophoresis. Additionally, further sequence validation of genomic rhLCV.eGFP BAC DNA isolated from bacteria was sequenced via PacBio sequencing, and the resulting genomic sequence deposited in GenBank under the accession number PX501811 (Table S2).

To generate a mutant gp350 rhLCV.eGFP BAC (rhLCV.eGFPΔgp350) devoid of gp350 protein expression, three-stop codons (5′-TAGTTAGATAGT-3′) were introduced into the gp350 gene of rhLCV.eGFP BAC between nucleotide positions 75,554 and 75,573 by *en passant* mutagenesis and screened as described above using the primers listed in Table S1. Further sequence validation of genomic rhLCV.eGFPΔgp350 BAC DNA isolated from bacteria was sequenced via Sanger sequencing (Fig. S4).

### Whole-genome rhLCV.eGFP BAC sequencing and sequence analysis

The PacBio third-generation sequencing platform was used to evaluate the integrity of rhLCV.eGFP BAC DNA isolated from GS1783 *E. coli*, as previously described (58). In brief, genomic DNA was purified using the Qiagen Plasmid Maxi Kit (Qiagen, Hilden, Germany), per the manufacturer’s protocol. Next, the fragmentation of 2 µg of isolated genomic DNA was conducted using the Covaris S220 ultrasonicator system (Matthew, NC), and the resulting sheared DNA sizes were analyzed using the Agilent 2011 bioanalyzer (Santa Clara, CA). SMRTbell libraries were constructed following the PacBio standard 20-kb template preparation protocol using the SMRTbell Template Prep Kit 1.0 from Pacific Biosciences (Menlo Park, CA). Briefly, the DNA was incubated with exonuclease VII (NEB) at 37°C for 15 min to remove single-stranded DNA, and any potentially damaged DNA was repaired using a DNA damage repair mix at 37°C for 20 min. Blunt-ended DNAs were treated with end-repair mix at 25°C for 5 min and ligated with 1 µM of annealed blunt adapters using 0.75 U/µL ligase at 25°C overnight; then, the ligase was inactivated by incubation at 65°C for 10 min. To remove failed ligation products, samples were treated with exonuclease III (NEB) and VII at 37°C for 1 h. To purify the DNAs and ligated products, 0.45× of AMPure PB Beads from Pacific Biosciences were applied. The final magbead complexes were loaded into a PacBio RSII machine for SMRT sequencing for a ~6 h runtime.

Primary whole-genome sequencing analysis, including real-time imaging, base calling, and assessing quality, was performed by the PacBio RS Blade Center through RS Touch and RS Remote, and the results were sent directly to secondary analysis for extracting the filtered subreads with SMRT Pipe (v.1.87.139483) via SMRT Portal (v.2.3.0). The filtered subreads were used as the input for the Canu sequence assembler (v.1.7.1) (88) for *de novo* assembly with options for PacBio HiFi reads, minimum reads of 5 kb, a minimum overlap length of 500 bp, and a minimum coverage of 5, and the assembled sequence from Canu was corrected using LoRDEC (v.0.9) (89), based on Illumina short reads obtained from the same DNA sample generated using an Illumina MiSeq (San Diego, CA). Then, minimap2 (v2.27) was adopted to align the HiFi long reads against both the reference genome and the *de novo* assembly.

### Reconstitution of rhLCV.eGFP and rhLCV.eGFPΔgp350 viruses and establishment of P3HR-1-based producer cell lines

To first reconstitute the recombinant rhLCV.eGFP viruses and establish virus-producer cell lines, we explored three different strategies:

#### Transfection of the γ-herpesvirus-free adherent cell lines C33A and HEK-293

C33A (or HEK-293) cells were seeded in six-well plates using their corresponding growing media (described above) at a 250,000 cells/well density. After 24 h, they were transfected with 5 µg of purified rhLCV.eGFP BAC DNA using polyethylenimine (PEI) transfection reagent at a 3:1 (DNA:PEI) ratio. After 48 h, the media were exchanged and supplemented with 1 µg/mL of puromycin. Media were changed every 48 h, until stable colonies were established and expanded. To test for lytic replication and viral production, the stable rhLCV.eGFP C33A (or HEK-293) cells were seeded in 150 mm dishes and, after 24 h, induced by transfection with rhLCV-SVN BRFL1 and/or rhLCV-SVN-BZLF1 plasmids (obtained from Dr. Frederick Wang of Brigham and Women’s Hospital, Harvard Medical School) in a medium with or without 20 ng/mL TPA and 3 mM NaB. One day post-induction, the media were removed and replaced with complete media without any antibiotics. On day 5 post-induction, the cell supernatants were collected, centrifuged at 4,000 × *g* for 20 min at 4°C, and filtered to remove cell debris (0.8 μm). The clarified supernatants were ultracentrifuged at 38,200 × *g* for 90 min at 4°C to concentrate the produced virus. The resulting pellets were resuspended in serum-free Opti-MEM, pooled, aliquoted, and stored at −80°C until use.

#### Generation of RTA and ZTA-inducible stable C33A and HEK-293 cell lines

A doxycycline-inducible expression system was purchased from Takara Biosciences Inc/Clontech (631338, Tet-On 3G Bidirectional inducible expression systems with mCherry), consisting of the regulator plasmid (pCMV-Tet3G) that contains a neomycin (G418) selection marker and a response plasmid (pTRE3G-BI-mCherry). We cloned rhLCV RTA and ZTA genes into the pTRE3G-BI-mCherry plasmid, interspaced with a self-cleavage 2A linker sequence (18 aa) (85) to overexpress the RTA and ZTA proteins. Sanger sequencing was used to verify the sequence fidelity of the cloned constructs. To generate doxycycline-inducible triple-stable rhLCV producer cell lines, we seeded 250,000 C33A (or HEK-293) cells per well in six-well plates 24 h before transfecting with 2 µg of pCMV-Tet3G, using PEI transfection reagent as described above. After 48 h, the cells were split into four 150 mm dishes without selection antibiotics, and 48 h after the transfer, the media were replaced with complete MEM (or DMEM) media supplemented with 1.2 mg/mL G418. Cells were cultured in G418-supplemented media with frequent medium changes until G418-resistant colonies appeared and expanded. After establishing pCMV-Tet3G-stable C33A and HEK-293 cell lines, the C33A (or HEK-293) stable cells were seeded in six-well plates at 250,000 cells/well. After 24 h, they were transfected with 2 µg of the response plasmid, pTRE3G-BI-mCherry-LCV RTA-2A-ZTA, and 100 ng of a linear hygromycin resistance gene expression cassette using PEI transfection reagent. At 48 h post-transfection, cells were split into four 150 mm plates using media without selection antibiotics. Spent media were replaced with media supplemented with 1.2 mg/mL G418 and 800 µg/mL hygromycin 48 h post-seeding and subsequently replaced every 48 h until drug-resistant colonies were formed. To screen for double-stable doxycycline-inducible cells, 50,000 of the transfected C33A (or HEK-293) cells were seeded per well in 48-well plates, and 48 h post-seeding, the cells were incubated with media supplemented with or without 1 µg/mL doxycycline. Expression of mCherry 48 h post-induction was used as an indicator of successful generation of the inducible double-stable C33A/HEK-293 cell lines.

Once the double-stable doxycycline-inducible C33A and HEK-293 cell lines were established, 250,000 C33A (or HEK-293) double-stable cells were seeded per well in 6-well plates 24 h before transfecting with 5 µg of purified rhLCV.eGFP BAC DNA using PEI transfection reagent, as previously described. After 48 h, cells were split into 150 mm dishes without selection antibiotics. After 48 h, the media were replaced with media supplemented with 1.2 mg/mL G418, 800 µg/mL hygromycin, and 1 µg/mL of puromycin. Media were changed every 48 h, until stable colonies were established and expanded. To screen for triple-stable doxycycline-inducible cells, the transfected C33A (or HEK-293) cells were seeded in 6-well plates at 250,000 cells/well, and 48 h post-seeding, the cells were incubated with media supplemented with or without 1 µg/mL doxycycline. Expression of mCherry and increased intensity of eGFP expression was used as an indicator of successful generation of triple-stable C33A and HEK-293 cell lines. The resulting cells were induced for rhLCV.eGFP lytic replication and viral production confirmation. Briefly, rhLCV.eGFP C33A (or HEK-293) triple-stable cells were expanded into 150 mm dishes under selection of 1.2 mg/mL G418, 800 µg/mL hygromycin, and 1 µg/mL of puromycin. Once 80% confluent, stable cells were induced with complete MEM (or DMEM) supplemented with 1 µg/mL of doxycycline in the absence of G418, hygromycin, and puromycin for 24 h. Subsequently, induction media were removed and replaced with complete MEM (or DMEM) media. On day 5 post-induction, supernatants were processed and stored as described above.

#### Transfection of the B cell lines P3HR-1 and BJAB

P3HR-1 (or BJAB) cells were transfected through electroporation with 2 µg of rhLCV.eGFP BAC. The cells were then cultured in selection media (complete RPMI 1640 supplemented with 100 µg/mL hygromycin B and 2 µg/mL puromycin) to establish stable cell lines. eGFP-expressing cells were enriched by FACS, as described below, and cultured in selection media; no BJAB cells were recovered after four rounds of attempts. After three rounds of sorting, rhLCV.eGFP P3HR-1 cells were expanded to 60 T-175 flasks in selection media and induced for lytic replication using induction media (complete RPMI 1640 containing 50 ng/mL TPA and 3 mM NaB) for 24 h. Following replacement of the induction media with complete RPMI 1640 media, the cells were further incubated for 96 h. The resulting supernatants were collected, purified, concentrated, and stored as described above. Following this successful strategy, the rhLCV.eGFPΔgp350 virus was similarly reconstituted and produced.

### Enrichment of eGFP-expressing cells by cell sorting

To enrich eGFP-expressing cells, cells in culture were gently resuspended and transferred into a 50 mL Falcon tube. An aliquot was used to assess cell count and viability using the TC20 automated cell counter (Bio-Rad, Hercules, CA), and the remaining cells were pelleted by centrifugation at 350 × *g* for 5 min. Pelleted cells were washed twice in 10 mL of 1% FBS in PBS and centrifuged at 350 × *g* for 5 min. Pelleted cells were gently resuspended in 3 mL of 1% FBS in PBS at a density of 10 million cells/mL and filtered through a 70 µm nylon mesh to eliminate clumps. The cells were sorted for eGFP expression using a BD FACSAria Fusion flow cytometer, BD Biosciences, Franklin Lakes, NJ. Sorted cells were collected in RPMI 1640 supplemented with 1% L-glutamine, 1% penicillin-streptomycin, 10% FBS, and 3 mM HEPES buffer. The sorted cells were centrifuged at 500 × *g* for 10 min and plated in six-well plates with selection media: RPMI 1640 supplemented with 1% L-glutamine, 1% penicillin-streptomycin, 20% FBS, 100 µg/mL hygromycin B, and 2 µg/mL puromycin. Cells were monitored and expanded gradually into T175 flasks.

### Generation, characterization, isotyping, and sequencing of anti-rhLCV gp350 antibodies

Anti-rhLCV gp350 antibodies were generated as reagents to characterize rhLCV.eGFP viruses using traditional mouse hybridoma methods previously established in our laboratory, with modifications (40). Briefly, two 8-week-old BALB/c mice were immunized intraperitoneally with ultraviolet-inactivated rhLCV (days 0, 14, 21, and 64), MVA-rhLCVgp350 (days 50 and 57), or purified His-tagged recombinant rhLCV gp350 ectodomain (day 78) obtained from GenScript Inc (Piscataway, NJ). Anti-rhLCV gp350-specific antibody titers in serially diluted mouse sera collected at days −3, 27, 71, and 84 (terminal bleed) were determined using indirect ELISA against immobilized rhLCV gp350 purified protein as described below. The two mice were euthanized on day 84, their splenocytes isolated, and the antibody-secreting B cells enriched by using a CD138-positive selection kit 18957 (STEMCELL Technologies, Vancouver, BC, Canada). Purified CD138-positive antibody-secreting cells were fused with P3 × 63Ag8.653 myeloma cells at a ratio of 1:1 in the presence of polyethylene glycol (Millipore Sigma). Fused cells were seeded in flat-bottom 96-well plates and selected in specialized hybridoma growth media with HAT (Thermo Fisher Scientific) and 10% FBS as described (90). Supernatants from the resulting clonal hybridomas were first screened by indirect ELISA, which revealed 20 positive clones that were then tested by immunoblot against purified rhLCV gp350 protein. The five clones that generated a positive signal in immunoblot were expanded stepwise from 96-well plates to T-25 flasks. At confluence in T-25 flasks, supernatants from individual clones were collected, clarified by centrifugation (4,000 × *g*, 10 min, 4°C), and filtered through a 0.22 μm filter. Antibodies were purified using protein G affinity chromatography, and their purities were analyzed by SDS-PAGE. Following buffer exchange to PBS and concentration using Amicon ultra-15 centrifugal filters Ultracel 10K (Millipore Sigma), the purified antibodies were quantified using NanoDrop (NanoDrop Technologies, Wilmington, DE). Further confirmatory ELISAs, immunoblots, and flow cytometry characterizations were performed using purified antibodies as described below. Purified anti-gp350 antibody 4A6 was subsequently isotyped using the mouse antibody isotyping kit (26179, Thermo Fisher Scientific), and the CDR sequence of the variable heavy chain and light chain (VL) of the antibody was determined by GenScript Inc (Table S2). The 4A6 CDR sequences were then cloned into an antibody expression vector and used to produce and purify recombinant 4A6 (r4A6, GenScript Inc).

### Characterization of anti-rhLCV gp350 antibodies

The newly generated anti-rhLCV gp350 antibodies were characterized using three different methods:

#### ELISA

Indirect ELISA was used to screen for positive hybridomas producing anti-rhLCV gp350 antibodies. In brief, 96-well immunoplates (Costar 3590; Corning Inc, Corning, NY) were coated with 50 µL of 0.5 µg/mL LCV gp350-His protein in PBS and incubated overnight at 4°C. After washing three times with 0.05% (vol/vol) Tween 20 1× PBS (PBST), the plates were blocked with 100 µL blocking buffer (3% [wt/vol] bovine serum albumin [BSA] in PBST) and incubated for 1 h while shaking at room temperature. After three washes with PBST, 100 µL of hybridoma supernatant or 10 µg/mL of purified antibodies were added to each well and incubated for 2 h with shaking at room temperature. The plates were washed three times, followed by a 1 h incubation with shaking at room temperature with 100 µL of HRP-conjugated goat anti-mouse IgG secondary antibody (1:2,000 in PBS; BioRad 1706516). The plates were washed three times, and 100 µL ABTS 2-component microwell peroxidase chromogenic substrate (2,2′-azino-bis 3-ethylbenzothiazoline-6-sulphonic acid; SeraCare Life Sciences, Milford, MA) was added. After 20 min, the reaction was stopped using 100 µL of 1× KPL peroxidase stop solution (SeraCare Life Sciences, Milford, MA). Absorbance was measured at 405 nm using a FilterMax F3 multi-mode microplate reader (Molecular Devices, San Jose, CA).

#### Flow cytometry

Characterization of anti-rhLCV gp350 antibodies via flow cytometry was performed on uninfected BHK-21 cells, BHK-21 cells infected with MVA, and BHK-21 cells infected with MVA-rhLCVgp350. Infected and mock-infected BHK-21 cells were harvested by scraping the cells from the plates, after which the cells were washed in PBS and pelleted via centrifugation at 350 × *g* for 5 min. Cells were resuspended in PBS and transferred to FACS tubes for incubation with primary antibodies (10 µg/mL) for 1 h at 4°C. Afterward, cells were washed twice with PBS, and each tube was incubated for 1 h with AF647-conjugated anti-mouse IgG at a 1:2,000 dilution in PBS. All stained cells were subsequently washed twice in PBS and fixed in 1% paraformaldehyde for 15 min at room temperature, before undergoing flow cytometry using the NovoCyte Quanteon, Agilent Technologies. Data analyses were performed using FlowJo Software (v10).

#### Immunoblot

To determine antibody binding specificity, BHK-21 cells were either uninfected or infected with MVA, MVA-rhLCVgp350, or MVA-rhLCVgp350.His were resuspended in mammalian protein extraction reagent (mPER) supplemented with 1 tablet of protease inhibitor/10 mL (Thermo Fisher Scientific), and incubated on ice for 1 h. The lysates were then pelleted by centrifugation at 15,000 × *g* for 15 min. Lysate supernatants were denatured at 95°C for 10 min in 1× Laemmli sample buffer and resolved on 12-well Bolt 4%–12% Bis-Tris Plus gels (Thermo Fisher Scientific). The gels were transferred onto iBlot 2 PVDF Mini Stacks (Thermo Fisher Scientific) membranes using the iBlot Transfer System (Thermo Fisher Scientific). Membranes were blocked for 1 h in blocking buffer (3% BSA in PBST) at room temperature. The membranes were subsequently incubated in blocking buffer containing 10 µg/mL purified anti-rhLCV gp350 antibodies or mouse anti-6×-His antibody (His.H8; 14-6657-82 Thermo Fisher Scientific) at a 1:1,000 dilution overnight at 4°C. After three washes with PBST, the membranes were incubated with a goat anti-mouse antibody conjugated to horseradish peroxidase (1:2,000 in blocking buffer; 1706516 BioRad) for 1 h. After three washes, the antibody-protein complexes were detected using the Amersham ECL Prime Western Blotting Detection Reagent from GE Healthcare, Chicago, IL, and images were captured using the Syngene PXi system. All experiments were independently repeated three times, and a representative blot is presented.

### gp350 detection in recombinant rhLCV.eGFP viruses by immunoblot

To confirm the presence of rhLCV gp350 protein in the recombinant viruses, the viruses were analyzed using SDS-PAGE, followed by immunoblot as described immediately above with two minor modifications. Aliquots of ultrapurified rhLCV.eGFP, rhLCV.eGFPΔgp350, and KSHV viruses were diluted in mPER at a 2:1 (virus:mPER) ratio and incubated on ice for 1 h. The resulting supernatants after centrifugation were resolved by SDS-PAGE and transferred to perform immunoblot. Second, during immunoblot, a recombinant version of the selected anti-rhLCV gp350 4A4 clone was used as primary antibody at 10 µg/mL in blocking buffer.

### Generation of EBV-free rhLCV.eGFP and rhLCV.eGFPΔgp350 producer cell lines

To generate rhLCV.eGFP and rhLCV.eGFPΔgp350 producer rhesus LCLs, PBMCs isolated from four rhLCV-naive rhesus macaques (33479, 33487, 33488, and 39754) were inoculated with purified P3HR1-rhLCV.eGFP or P3HR1-rhLCV.eGFPΔgp350 and cultured for 6–8 weeks until transformation. Infected eGFP-positive B cells were sorted as described in “Enrichment of eGFP-expressing cells by cell sorting,” above, periodically during the cell line establishment. eGFP-enriched cells were expanded and induced for lytic replication as described for P3HR-1 with a minor modification: induced cells were maintained in induction media, and no media exchange was performed until harvesting.

### Titration of rhLCV.eGFP and rhLCV.eGFPΔgp350 virus stocks

rhLCV.eGFP and rhLCV.eGFPΔgp350 were titrated in Raji cells to determine the Raji infectious units (RIU) per volume in each virus stock. Raji cells were seeded in triplicate at a cell density of 50,000 cells per well in a 96-well plate. Cells were incubated at 37°C for 1 h with varying amounts of virus diluted in serum-free Opti-MEM in a total volume of 100 µL per well. After which, 200 µL of complete media was added, and the cells were cultured for 24 h. Cells were then harvested and collected into FACS tubes or U-bottom 96-well FACS plates and centrifuged at 500 × *g* for 5 min. The cell pellets were then washed three times with cell staining buffer (DPBS with 0.5% BSA and 2 mM EDTA) and subsequently resuspended and fixed in 1% paraformaldehyde for 15 min at room temperature prior to analysis on the NovoCyte Quanteon. Data analyses were performed using FlowJo Software (v10) to obtain the percentage of eGFP+ cells in each condition. To calculate RIU per volume, the following formula described in (91) was applied to a single representative point value on the linear phase of the curve: RIU/volume of virus = (number of cells at time of infection × percentage of eGFP + cells)/(volume of inoculum).

### Viral DNA/RNA detection by qPCR

DNA extraction from cells and viruses was conducted using the MagMAX DNA Multi-Sample Ultra 2.0 Kit (A45721, Thermo Fisher Scientific) on the Kingfisher Duo Primer (5400110, Thermo Fisher Scientific) following the manufacturer’s instructions. To quantify genomic virus DNA from purified viruses, aliquots were pre-incubated with DNase I at 0.1 U/µL (EN0521, Thermo Fisher Scientific) at 37°C for 1–2 h, followed by denaturation at 75°C for up to 1 h, before DNA extraction.

RNA extraction from induced or uninduced LCLs was conducted using the MagMAX mirVana Total RNA Isolation Kit (A27828, Thermo Fisher Scientific) on the Kingfisher Duo Primer following the manufacturer’s instructions. Subsequently, cDNA was synthesized from 100 to 300 ng RNA using SuperScript IV VILO Kit (11766050, Thermo Fisher Scientific) following the manufacturer’s instructions.

qPCRs were conducted on undiluted DNA or cDNA adjusted to 25 ng/test on the QuantStudio 3 or QuantStudio 7 Pro Real-Time PCR Systems. Each reaction was set to 20 µL, comprising 1 µL of the respective primer pair (each primer at 600 nM), 10 µL PowerUp SYBR Green Master Mix (A25741, Thermo Fisher Scientific), 4 µL nuclease-free water, and 5 µL DNA or cDNA sample. Amplification conditions were as follows: initial denaturation at 95°C for 2 min, followed by 45 cycles of 95°C for 15 s and 60°C for 1 min. After amplification completion, melting curves were obtained by increasing 1.6°C/s from 65°C to 95°C. Standard curves were constructed by a 1:10 serial dilution (10^7^ to 1 copy/µL) of gBlocks comprising DNA fragments of rhLCV EBER, rhLCV LMP2B, or EBV BALF5 (Table S4) for their respective genomic viral DNA quantification. The results were analyzed using the QuantStudio Design and Analysis Software v2.6.0, Thermo Fisher Scientific.

### Viral neutralization assays

Akata-EBV-eGFP virus, rhLCV.eGFP, and rhLCV.eGFPΔgp350 were pre-incubated with 25 µg of anti-EBV gp350 [72A1] or anti-EBV gHgL [AMMO1], each in a total of 60 µL in Opti-MEM, for 1 h. In parallel, Akata-4E3, BJAB, or Raji cells were seeded at 50,000 cells/well in 50 µL of Opti-MEM. Next, 50 µL of the virus-antibody mixture were added to the seeded cells and further incubated for 1 h, after which 150 µL of complete RPMI 1640 media was added to each well. Non-antibody-treated infected and uninfected cells were used as positive and negative controls, respectively. Cells were analyzed 24 h post-inoculation by microscopy, and eGFP+ cells were quantified by flow cytometry using the method described in “Titration of rhLCV.eGFP and rhLCV.eGFPΔgp350 virus stocks,” above. The obtained eGFP+ % values were used to estimate the % neutralization as the percentual eGFP+ reduction in antibody-treated conditions vs. the respective positive control. All determinations were performed in triplicate.

### Negative staining transmission electron microscopy

Formvar/Carbon copper grids (200 mesh, TedPella Inc) were glow-discharged for 20 s, and then, 5 µL of eGFP-expressing viruses, previously fixed with 4% paraformaldehyde for 5 min at 0°C, was applied and incubated for 1 min at room temperature. Grids were rinsed three times in Milli-Q water (20 s each), blotted, and stained with 5 µL of 1% uranyl acetate for 1 min. Excess stain was removed, and grids were air-dried. Images were acquired using a FEI Tecnai 12 Twin TEM operating at 120 keV equipped with a Gatan 894 Ultrascan 1000 CCD camera.

### *In vitro* and *ex* vivo infection assays

To determine the susceptibility of various cell types to rhLCV.eGFP and rhLCV.eGFPΔgp350 infection, we inoculated a diverse array of cells from different species, including rhesus macaque cell lines (RF-6A, DBS-FRhL-2, LLC-MK2, 4MBr-5, and LCL 8664), human cell lines (C33A, HFF-1, HEK 293, HMVEC-dAd, HeLa, Akata-4E3, BC1, BC-2, BC-3, BCBL-1, BJAB, P3HR-1, and Raji), non-human primate cell lines (AG23 AGM BLCL, V038 AGM BLCL, S594, and Vero), and PBMCs from both human and non-human primates (chimpanzee, baboon, rhesus macaque, cynomolgus macaque, pigtail macaque, common marmoset, and capuchin monkey). All adherent cells were seeded at a density of 75,000 cells/well in a 48-well plate and cultured for 24 h. After aspirating the spent media, the cells were infected with either 1.2 × 10^4^ RIU (RF-6A, DBS-FRhL-2, LLC-MK2, and 4MBr-5) or 6 × 10^4^ RIU (C33A, HFF-1, HEK 293, HMVEC-dAd, HeLa, and Vero) of LCL1-rhLCV.eGFP or LCL4-rhLCV.eGFPΔgp350 viruses in 100 µL Opti-MEM. Following a 1 h incubation at 37°C, 200 µL fresh media were added. B cell lines and PBMCs were seeded at 50,000 cells/well and 200,000 cells/well, respectively, in 96-well plates, and inoculated with either 2 × 10^5^ RIU of P3HR1-rhLCVeGFP (or P3HR1-rhLCVeGFP Δgp350) or 1.2 × 10^4^ RIU of LCL1-rhLCV.eGFP (or LCL4-rhLCV.eGFPΔgp350) viruses, unless otherwise noted.

Microscopic imaging of eGFP expression was captured 24 h post-infection, after which the cells were harvested. Harvested cells were alternatively used for eGFP quantification via flow cytometry as described in “Titration of rhLCV.eGFP and rhLCV.eGFPΔgp350 virus stocks,” above, or processed to assess the presence of viral DNA as described in “Viral DNA/RNA detection by qPCR,” above. Uninfected cells of each cell type served as mock virus controls.

To determine the virus transformation capacity, LCL1-rhLCV.eGFP-inoculated rhesus macaque PBMCs (33470 and 33483) were periodically monitored by microscopic imaging until achieving transformation and stable eGFP expression (28–39 days).

## Data Availability

The whole-genome sequence of rhLCV.eGFP BAC has been submitted to GenBank under accession number PX501811.

## SUPPLEMENTAL MATERIAL

Supplemental material jvi.00537-26-s0001.pdf Tables S1 to S4; Fig. S1 to S9.

ASM does not own the copyrights to Supplemental Material that may be linked to, or accessed through, an article. The authors have granted ASM a non-exclusive, world-wide license to publish the Supplemental Material files. Please contact the corresponding author directly for reuse.
