# Supplementary material for: A newly developed eGFP-traceable recombinant rhesus lymphocryptovirus, a tool to study viral infection and replication in vitro and ex vivo, confirms gp350 as key for viral entry
Source: J Virol. 2026 Jun 11;100(7):e00537-26. doi: 10.1128/jvi.00537-26 (PMC13386976; doi:10.1128/jvi.00537-26)
Supplement: Supplemental material — Tables S1 to S4; Fig. S1 to S9. [file jvi.00537-26-s0001.pdf]

Table S1. Primers for generation and characterization of rhLCV.eGFP and rhLCV.eGFPΔgp350 BAC constructs.

Purpose	Sequence (5’-3’)
eGFP-CmR-PuroR *en passant* PCR	CTGGCTGCTAGCCACTCGCCACACGTGGTGCCAGTCCCAGCATTTCCACACCGCGTTACATAACTTACGGTAAATG
	ACCCCAGTAGGCACAAGGGTGCCTCGTGGATGAGACAATGGAAAGGTGTTGTATCGATAAGCTTGATCGCG
eGFP-CmR-PuroR *en passant* colony PCR + eGFP-PuroR PCR for Sanger sequencing	TGATGTCCTGTCTCTGAGTG
	TCCACTTCTCCTGTGTAAC
eGFP-PuroR Sanger sequencing	TGATGTCCTGTCTCTGAGTG
	TCCACTTCTCCTGTGTAAC
	TGGCGTTACTATGGGAACATACG
	TACGGCGTGCAGTGCTTCAG
	TGGTAGTGGTCGGCGAGCT
	CTGCAAGAACTCTTCCTCAC
	TTCGGGCACCTCGACGT
3-stop element-CmR *en passant* PCR*	GGAAAACACTCTGGAGGGTGTATTGACATAACAGAAAAGCCTATCTAACTACGCTTC
	AAGGGGCTGTTTGCGAATCTGCACTGAAACAATGGAAGCGTAGTTAGATAGGCTTTT
3-stop element-CmR *en passant* colony PCR	TTGTCTGCAACAAAAGCATGACTCACCAAC
	TGAGGGAACACGACCGCGAGAAGCCGCG
3-stop element PCR for Sanger sequencing	TTGTCTGCAACAAAAGCATGACTCACCAAC
	GCACGTGAAATAATTAGTGTTTTCGAGG
3-stop element Sanger sequencing	AGTGCAGACCTCACACTTG

**3-stop element underlined*

Table S2: BLAST analysis of rhLCV.eGFP BAC whole-genome sequence (GenBank: PX501811)									
#	Coding sequence	Query coverage	Identity to reference genome (NC_006146)	E Value	Hit start	Hit end	Length (nt)	Strand	Description
1	LMP2B	22.19%	99.60%	2.30E-126	644	892	249	+	Terminal protein LMP2B
2	BNRF1	100.00%	100.00%	0	1836	5780	3945	+	FGAM-synthase
3	BCRF1	100.00%	100.00%	0	10163	10696	534	+	TBP-like protein BcRF1
4	EBNA-LP	12.63%	97.90%	5.06E-65	15403	15543	141	+	EBV nuclear antigen leader protein
5	EBNA-2	100.00%	100.00%	0	27791	29608	1818	+	Nuclear antigen EBNA-2
6	BHRF1	100.00%	100.00%	0	33700	34275	576	+	Apoptosis regulator BHRF1
7	BFLF2	100.00%	100.00%	0	36317	35364	954	-	Virion egress protein BFLF2
8	BFLF1	100.00%	100.00%	0	37904	36330	1575	-	Packaging protein UL32
9	BFRF1	100.00%	100.00%	0	38264	39253	990	+	Virion egress protein UL34 homolog
10	BFRF2	100.00%	100.00%	0	39157	40968	1812	+	Late gene expression regulator BFRF2
11	BFRF3	100.00%	100.00%	0	40892	41404	513	+	Small capsomere-interacting protein
12	BPLF1	100.00%	99.40%	0	50801	41445	9361	-	Large tegument protein
13	BORF 1	100.00%	100.00%	0	54536	55627	1092	+	Capsid triplex subunit 1
14	BOLF1	100.00%	100.00%	0	54537	50851	3687	-	Inner tegument protein
15	BORF2	100.00%	100.00%	0	55689	58157	2469	+	Ribonucleoside-diphosphate reductase
16	BaRF1	100.00%	100.00%	0	58170	59078	909	+	Ribonucleoside-diphosphate reductase
17	BMRF1	100.00%	99.80%	0	59171	60382	1215	+	DNA polymerase processivity factor
18	BMRF2	100.00%	100.00%	0	60387	61460	1074	+	BMRF2 protein
19	BMLF1	100.00%	100.00%	0	65664	64267	1398	-	mRNA export factor ICP27
20	BSLF1	100.00%	100.00%	0	68485	65861	2625	-	Helicase-primase primase complex protein
21	BSRF1	100.00%	100.00%	0	68528	69193	666	+	Tegument protein UL51 homolog
22	BLLF3	100.00%	99.90%	0	70095	69256	840	-	dUTPase
23	BLRF1	100.00%	100.00%	3.82E-161	70169	70477	309	+	Glycoprotein N
24	BLRF2	100.00%	100.00%	0	70548	71039	492	+	Tegument protein BLRF2
25	BLLF2	100.00%	100.00%	0	71615	71196	420	-	BLLF2 protein
26	BLLF1	100.00%	100.00%	0	73407	71056	2352	-	Glycoprotein 350
27	EBNA-3A	89.00%	100.00%	0	73938	76452	2515	+	Nuclear antigen EBNA-3A
28	EBNA-3B	86.90%	99.80%	0	77080	79501	2423	+	Nuclear antigen EBNA-3B
29	EBNA-3C	90.11%	99.60%	0	80055	83187	3136	+	Nuclear antigen EBNA-3C
30	BZLF2	100.00%	100.00%	0	83900	83235	666	-	Glycoprotein 42
31	BZLF1	68.95%	100.00%	0	85088	84576	513	-	Viral immediatly early antigen
32	BRRF1	100.00%	100.00%	0	87100	88029	930	+	Transcriptional activator BRRF1
33	BRLF1	100.00%	99.60%	0	87101	85296	1810	-	Replication and transcription activator
34	BRRF2	100.00%	100.00%	0	88218	89723	1506	+	Tegument protein BRRF2
35	EBNA-1	100.00%	100.00%	0	89759	91294	1536	+	Nuclear antigen EBNA-1
36	BKRF2	100.00%	100.00%	0	91375	91788	414	+	Envelope glycoprotein L
37	BKRF3	100.00%	100.00%	0	91770	92537	768	+	Uracil-DNA glycosylase
38	BKRF4	100.00%	100.00%	0	92548	93267	720	+	Tegument protein BKRF4
39	BBRF1	100.00%	100.00%	0	95683	97527	1845	+	Virion protein BBRF1
40	BBLF4	100.00%	100.00%	0	95738	93309	2430	-	Helicase-primase subunit

Table S2: BLAST analysis of rhLCV.eGFP BAC whole-genome sequence (GenBank: PX501811) - *continuation*									
#	Coding sequence	Query coverage	Identity to reference genome (NC_006146)	E Value	Hit start	Hit end	Length (nt)	Strand	Description
41	BBRF2	100.00%	97.40%	0	97430	98266	837	+	Cytoplasmic envelopment protein 1
42	BBLF3	99.83%	92.20%	0	98837	98263	575	-	Helicase complex BBLF3
43	BBLF2	100.00%	97.90%	0	100541	98937	1609	-	Helicase complex BBLF2
44	BBRF3	100.00%	98.60%	0	100641	101861	1221	+	Envelope glycoprotein M
45	BBLF1	100.00%	100.00%	1.08E-115	102584	102360	225	-	Alkaline exonuclease, UL11/BBLF1 tegument protein
46	BGLF5	100.00%	98.40%	0	103951	102539	1413	-	Shutoff alkaline exonuclease
47	BGLF4	100.00%	99.50%	0	105227	103938	1290	-	Serine/threonine-protein kinase BGLF4
48	BGLF3	100.00%	99.80%	0	106552	105554	999	-	Late gene expression regulator BGLF3
49	BGLF2	100.00%	99.80%	0	108486	107476	1011	-	Cytoplasmic envelopment protein 2
50	BGLF1	100.00%	94.00%	0	109960	108464	1497	-	BGLF1 protein
51	BDLF4	100.00%	96.30%	0	110652	109930	723	-	Late gene expression regulator BDLF4
52	BDRF1	54.85%	98.10%	0	110849	111985	1137	+	BDRF1 protein
53	BDLF3	100.00%	100.00%	0	112775	111996	780	-	Glycoprotein BDLF3
54	BDLF2	100.00%	100.00%	0	114051	112840	1212	-	Protein BDLF2
55	BDLF1	100.00%	100.00%	0	114966	114061	906	-	Capsid triplex subunit 2
56	BcLF1	100.00%	100.00%	0	119124	114982	4143	-	Major capsid protein
57	BcRF1.1	100.00%	99.90%	0	119666	121399	1734	+	Envelope protein
58	BTRF1	100.00%	100.00%	0	121386	122597	1212	+	Tegument protein
59	BXLF2	100.00%	99.80%	0	124714	122594	2124	-	Envelope glycoprotein H
60	BXRF1	100.00%	99.10%	0	126538	127287	750	+	Protein UL24 homolog
61	BXLF1	100.00%	99.70%	0	126539	124716	1824	-	Thymidine kinase
62	BVRF1	100.00%	98.90%	0	127097	128803	1707	+	Virion protein BVRF1
63	BVRF2	100.00%	99.90%	0	129611	131470	1860	+	Capsid scaffolding protein
64	BILF2	100.00%	100.00%	0	132265	131504	762	-	Glycoprotein BILF2
65	LF3	90.72%	99.00%	0	137575	135176	2425	-	LF3 protein
66	LF2	100.00%	99.90%	0	146186	144897	1290	-	LF2 protein
67	LF1	100.00%	100.00%	0	147541	146147	1395	-	LF1 protein
68	BILF1	100.00%	100.00%	0	148488	147550	939	-	G-protein coupled receptor BILF1
69	ECRF4	100.00%	99.80%	0	150614	151750	1137	+	ECRF4 protein
70	BALF5	100.00%	99.90%	0	152134	149087	3048	-	DNA polymerase (EC 2.7.7.7)
71	BALF4	100.00%	100.00%	0	154731	152137	2595	-	Glycoprotein B
72	BARF0	100.00%	100.00%	0	155921	156406	486	+	BARF0 protein
73	BALF3	100.00%	100.00%	0	156772	154718	2055	-	Tripartite terminase subunit 1
74	BALF2	100.00%	99.40%	0	160177	156788	3402	-	Single-stranded DNA binding protein
75	BALF1	100.00%	100.00%	0	160810	160262	549	-	Apoptosis regulator BALF1
76	BARF1	100.00%	100.00%	0	160910	161572	663	+	CSF1R homologue
77	LMP2a	24.24%	100.00%	0	171949	172308	360	+	LMP2a protein
78	BNLF2b	100.00%	100.00%	1.20E-154	172684	172388	297	-	BNLF2B protein
79	BNLF2a	100.00%	100.00%	2.29E-91	172872	172693	180	-	BNLF2a protein
80	LMP1	80.40%	98.70%	0	175135	173731	1423	-	Latent membrane protein 1 p63

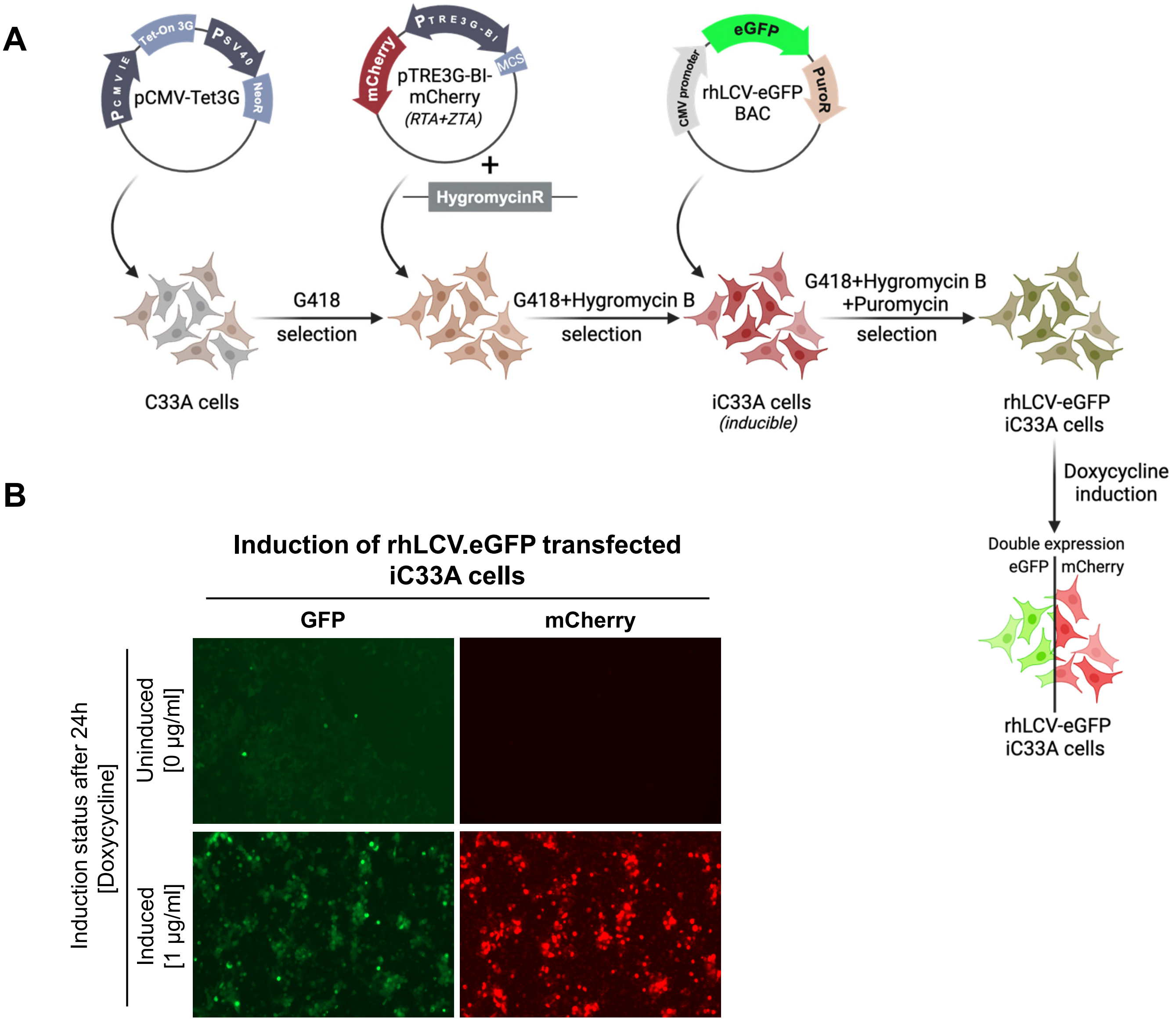
A
PCMV IE
Tet-On 3G
PSV40
NeoR
pCMV-Tet3G
PTRE3G-BI
mCherry
MCS
pTRE3G-BI-mCherry
(RTA+ZTA)
+
HygromycinR
eGFP
CMV promoter
PuroR
rhLCV-eGFP BAC
C33A cells
G418 selection
G418+Hygromycin B selection
iC33A cells
(inducible)
G418+Hygromycin B +Puromycin selection
rhLCV-eGFP iC33A cells
Doxycycline induction
Double expression
eGFP
mCherry
rhLCV-eGFP iC33A cells
B
Induction of rhLCV.eGFP transfected iC33A cells
GFP
mCherry
Induction status after 24h [Doxycycline]
Uninduced [0 µg/ml]
Induced [1 µg/ml]

Fig S1. Generation of a doxycycline-inducible C33A rhLCV.eGFP-producer cell line. (**A**) Schematic diagram illustrating the generation of doxycycline-inducible C33A rhLCV.eGFP-producer cells under a Tet On system via sequential transfections. C33A cells were first transfected with the Tet regulator plasmid pCMV-Tet3G, which contains a neomycin resistance gene; cells were cultured under selection with G418. The resulting pCMV-Tet3-stable cells were transfected with a Tet response, pTRE3G-BI-mCherry, that had been cloned with a bicistronic expression cassette coding for rhLCV RTA and ZTA, together with a linear expression cassette coding for the hygromycin resistance gene; cells were subsequently cultured under both G418 and hygromycin selection. The resulting iC33A double-stable cells were then transfected with rhLCV.eGFP BAC DNA, and subsequently cultured under G418, hygromycin, and puromycin selection. This resulted in rhLCV.eGFP iC33A triple-stable cells, which under doxycycline induction are expected to express eGFP, mCherry, and the rhLCV RTA and ZTA proteins. (**B**) Induction of rhLCV.eGFP iC33A cells. rhLCV.eGFP iC33A cells generated as in (A) were induced by incubation in doxycycline (1μg/ml)-containing media for 24 h. Shown are GFP and mCherry channel micrographs of the cells before (top row) and after (bottom row) 24 h of induction.

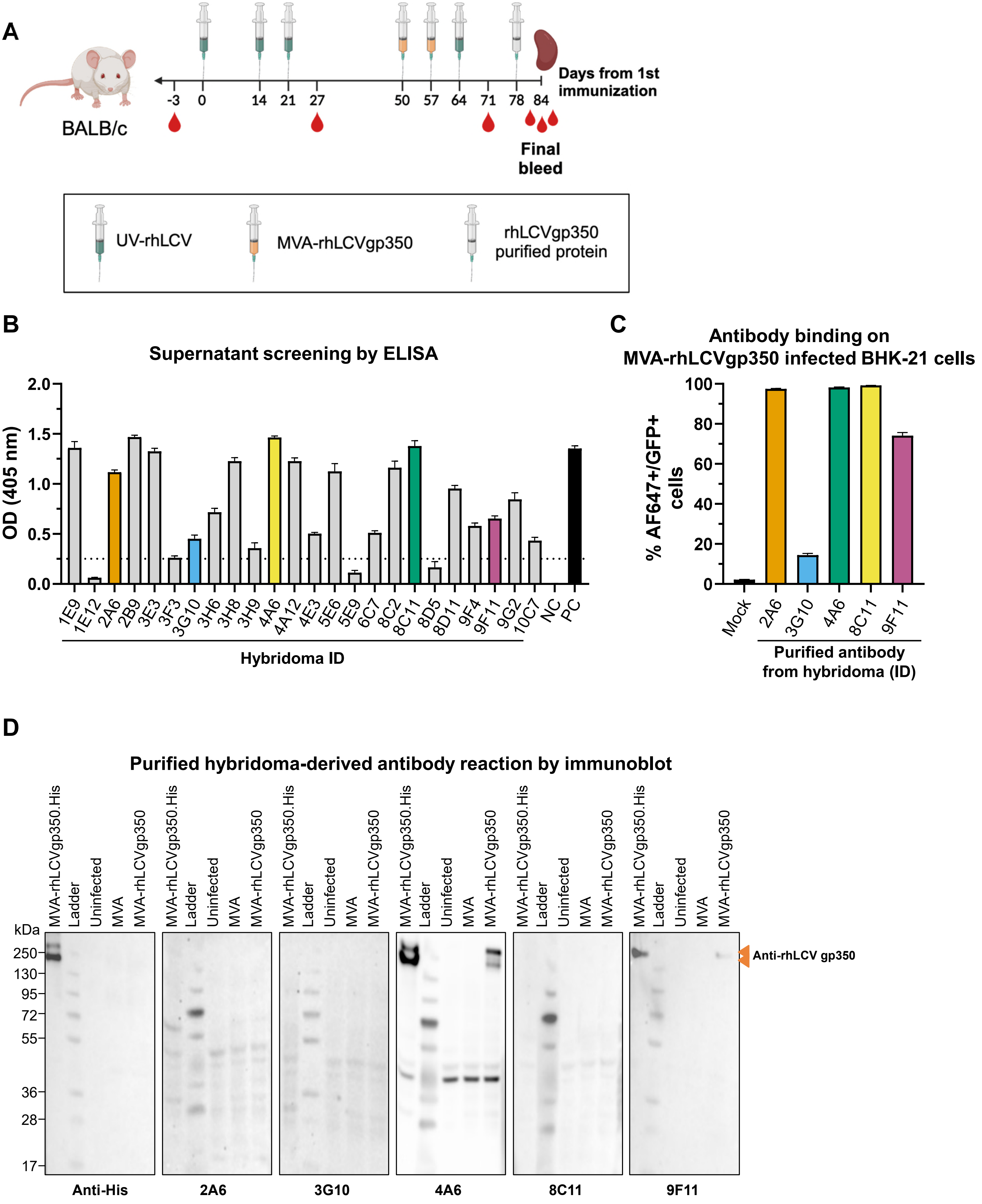

A
BALB/c
-3
0
14
21
27
50
57
64
71
78
84
Days from 1st immunization
Final bleed
UV-rhLCV
MVA-rhLCVgp350
rhLCVgp350 purified protein
B
Supernatant screening by ELISA
OD (405 nm)
2.0
1.5
1.0
0.5
0.0
1E9
1E12
2A6
2B9
3E3
3F3
3G10
3H6
3H8
3H9
4A6
4A12
4E3
5E6
5E9
6C7
8C2
8C11
8D5
8D11
9F4
9F11
9G2
10C7
NC
PC
Hybridoma ID
C
Antibody binding on MVA-rhLCVgp350 infected BHK-21 cells
% AF647+/GFP+ cells
100
80
60
40
20
0
Mock
2A6
3G10
4A6
8C11
9F11
Purified antibody from hybridoma (ID)
D
Purified hybridoma-derived antibody reaction by immunoblot
MVA-rhLCVgp350.His
Ladder
Uninfected
MVA
MVA-rhLCVgp350
kDa
250
130
95
72
55
36
28
17
Anti-rhLCV gp350
Anti-His
2A6
3G10
4A6
8C11
9F11

Fig S2. Generation and characterization of anti-rhLCV gp350 antibodies. (**A**) Schematic diagram of the BALB/c mice (n=2) immunization schedule used to generate rhLCV-gp350 murine antibodies. (**B**) ELISA screening of hybridoma supernatants to detect anti-rhLCV gp350 antibodies. Collected spleens from mice in (A) were used to generate hybridomas, and resulting hybridoma culture supernatants were screened by ELISA for the presence of anti-rhCLV gp350 antibodies, using soluble rhLCV gp350 protein as the target antigen, and horseradish peroxidase-conjugated anti-mouse as secondary antibody. Supernatants from hybridomas shown in color, which generated a positive signal in immunoblot against purified gp350 protein (not shown), were subsequently purified via protein G affinity chromatography, and further characterized as described in (C) and (D). (**C**) Flow cytometric assessment of purified hybridoma supernatant IgG against rhCLV gp350. BHK-21 cells infected with gp350-expressing MVA virus were harvested and processed for flow cytometry, using the listed purified antibodies as primary antibody, and AF647-conjugated anti-mouse antibody as secondary antibody. Unstained cells were used as a mock control. (**D**) Immunoblot assessment of purified hybridoma supernatant IgG against rhCLV gp350. The listed purified antibodies were used as primary antibodies in immunoblot assay against lysates of BHK-21 cells infected with MVA expressing rhLCV gp350 and gB (MVA-rhLCVgp350) or His-tagged rhLCV gp350 (MVA-rhLCVgp350.His), using horseradish peroxidase-conjugated anti-mouse antibody as secondary antibody. Uninfected and MVA-infected BHK-21 cell lysates were used as negative controls. Expected protein sizes are indicated with orange arrows.

Table S3. Isotype and CDR sequences of anti-rhLCV gp350 antibody, 4A6				
**Chain**	**Isotypes**	**CDR sequences**		
		**CDR1**	**CDR2**	**CDR3**
Heavy	IgG1	SDYAWN	CITYSGSTSYSPSLKS	SNYVNYFDY
Light	Kappa	KSSQSLLYSSNQKNYLA	WASTRES	QQYYDYPT

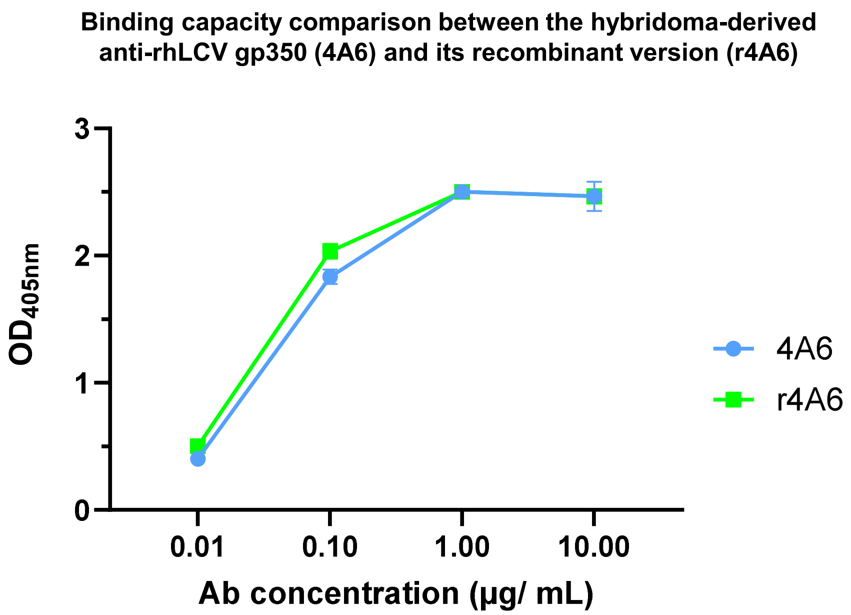

Binding capacity comparison between the hybridoma-derived anti-rhLCV gp350 (4A6) and its recombinant version (r4A6)
3
2
1
0
OD405nm
0.01
0.10
1.00
10.00
Ab concentration (μg/ mL)
4A6
r4A6

Figure S3. Binding capacity assessment of the recombinant anti-rhLCV gp350 [4A6]. The binding capacity of the recombinant anti-rhLCV gp350 [4A6] (r4A6) was compared to the parental hybridoma-derived 4A6 purified antibody by ELISA, using soluble rhLCV gp350 protein as the target antigen, and horseradish peroxidase-conjugated anti-mouse as secondary antibody. Both r4A6 and 4A6 were serially diluted and tested at concentrations ranging from 10 µg/mL to 0.01 µg/mL. Reactions were measured by spectrophotometry at an absorbance of 405 nm with dilution buffer blank correction.

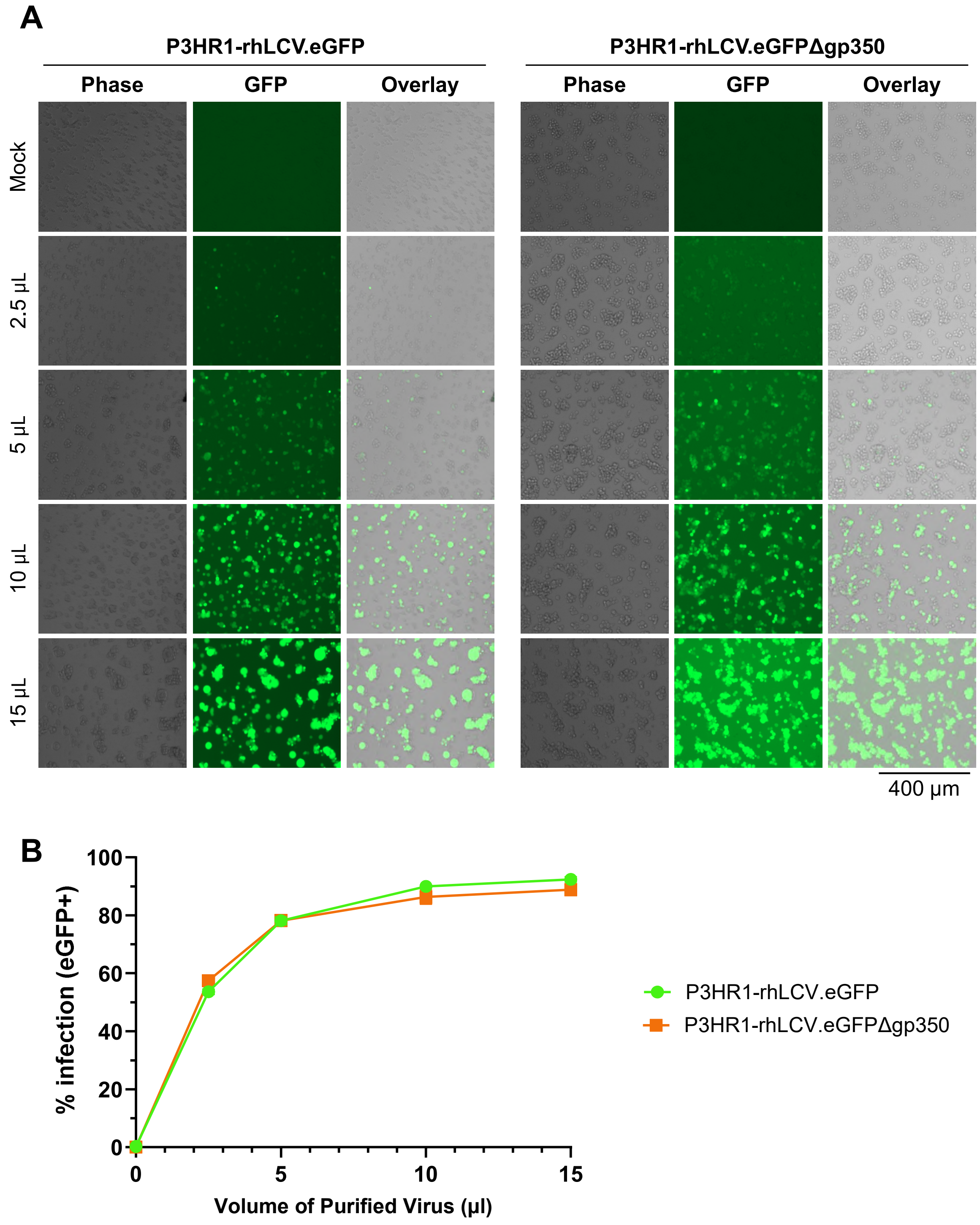
A
P3HR1-rhLCV.eGFP
P3HR1-rhLCV.eGFPΔgp350
Phase
GFP
Overlay
Mock
2.5 µL
5 µL
10 µL
15 µL
400 µm
B
% infection (eGFP+)
Volume of Purified Virus (µl)
0
20
40
60
80
100
0
5
10
15
P3HR1-rhLCV.eGFP
P3HR1-rhLCV.eGFPΔgp350

Figure S4. P3HR1-rhLCV.eGFP and P3HR1-rhLCV.eGFPΔgp350 titration on Raji cells. (**A**) Microscopic analysis of Raji cells infected with different volumes of purified P3HR1-rhLCV.eGFP or P3HR1-rhLCV.eGFPΔgp350 viruses. Raji cells were inoculated with the listed volumes of P3HR1-rhLCV.eGFP or P3HR1-rhLCV.eGFPΔgp350 viruses and incubated for 24 h. Shown are representative Phase, GFP, and phase-GFP-channel-merged micrographs of triplicate samples for each inoculum volume 24 h after infection. Uninfected cells were used as a mock control. (**B**) Flow cytometry analysis of Raji cells infected with different volumes of purified P3HR1-rhLCV.eGFP or P3HR1-rhLCV.eGFPΔgp350 viruses. Cells from (A) were harvested and processed for flow cytometry analysis of eGFP expression. Shown is the mean + SEM quantification of infected (eGFP-expressing) cells at each volume for triplicates, which was used to calculate the RIU/volume for each virus as described in Methods.

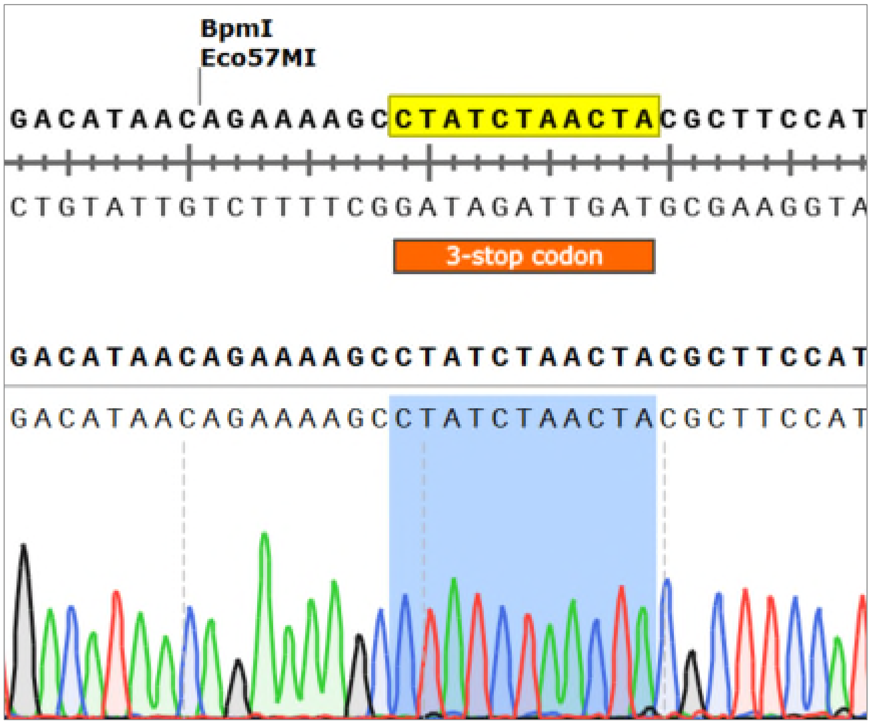

rhLCV.eGFPΔgp350 3' - G A C A T A A C A G A A A A G C C T A T C T A A C T A C G C T T C C A T - 5'

rhLCV.eGFP 3' - G A C A T A A C A G A A A A G C - - - - - - - - - - - C G C T T C C A T - 5'

Fig S5. Sanger sequencing verification of inserted three-stop codon element in rhLCV.eGFPΔgp350 BAC DNA. DNA was isolated from GS1783 bacteria harboring the rhLCV.eGFPΔgp350 BAC DNA after *en passant* mutagenesis. Obtained DNA was used as a template to PCR-amplify the three-stop codon element insertion site, and the resulting amplicons were analyzed by Sanger sequencing. Obtained sequencing reads were aligned to the rhLCV.eGFPΔgp350 expected genome using SnapGene software. Shown is a screenshot of the alignment confirming the presence of the three-stop codon element, as compared to the intact rhLCV.eGFP genome sequence.

A

Category	DNA source	qPCR targets and outcomes			
		**EBV** BALF5	**EBV** LMP2B	**rhLCV** EBER	**rhLCV** LMP2B
Cells	P3HR-1 *(EBV+/rhLCV- control)*	☑	☑	☒	☒
	LCL 8664 *(EBV-/rhLCV+ control)*	☒	☒	☑	☑
	rhLCV.eGFP infected P3HR1	☑	☑	☑	☑
	rhLCV.eGFPΔgp350 infected P3HR1	☑	☑	☑	☑
	BJAB *(EBV-/rhLCV- control)*	☒	☒	☒	☒
Viruses	P3HR1-rhLCV.eGFP	☑	☑	☑	☑
	P3HR1-rhLCV.eGFPΔgp350	☑	☑	☑	☑
	Akata-EBV-eGFP	☑	☑	☒	☒
Plasmid	rhLCV.eGFP BAC	☒	☒	☑	☑
Synthetic DNA	EBV BALF5 gBlock	☑	☑	☒	☒

B

Category	DNA source	qPCR targets and outcomes		
		**EBV** BALF5	**rhLCV** EBER	**rhLCV** LMP2B
Infected cells	Uninfected BJAB *(EBV-/rhLCV- control)*	☒	☒	☒
	rhLCV.eGFP infected BJAB	☑	☑	☑
	rhLCV.eGFPΔgp350 infected BJAB	☑	☑	☑
Plasmid	rhLCV.eGFP BAC	☒	☑	☑
Synthetic DNA	EBV BALF5 gBlock	☑	☒	☒

C

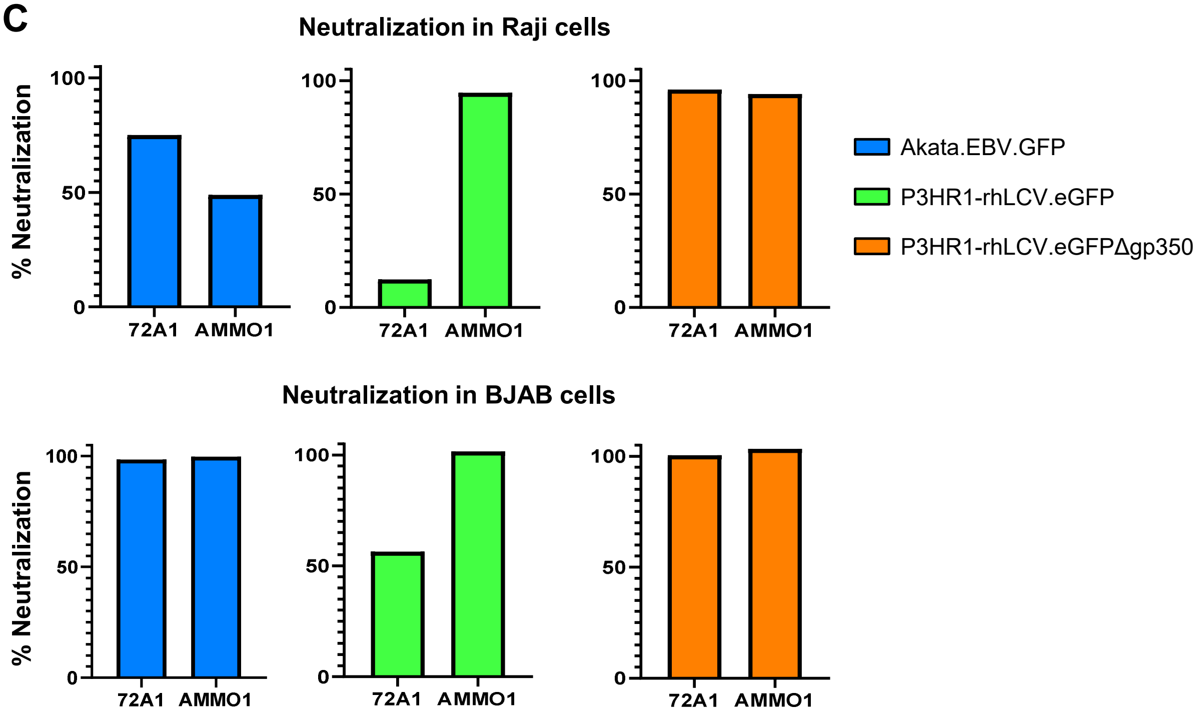

Figure S6. Assessment of EBV genomic presence and viral infection complementation in the purified P3HR1-rhLCV.eGFP and P3HR1-rhLCV.eGFPΔgp350 viruses. (**A**) EBV genomic presence determination in P3HR1-rhLCV.eGFP and P3HR1-rhLCV.eGFPΔgp350 producer cell lines and purified viruses. DNA was extracted from all indicated cell lines and purified viruses, and SYBR green-based qPCR performed to detect genomic EBV or rhLCV DNA using EBV LMP2B-, EBV BALF5-, rhLCV EBER- and rhLCV LMP2B-specific primers (Table S4). rhLCV.eGFP BAC plasmid and EBV BALF5 gBlock (Table S4) were amplified as additional rhLCV and EBV positive controls, respectively. Green ticks (☑) and red crosses (☒) represent detectable and undetectable target DNA, respectively. (**B**) EBV genomic presence determination in P3HR1-rhLCV.eGFP and P3HR1-rhLCV.eGFPΔgp350 infected BJAB cells. BJAB cells were inoculated with P3HR1-rhLCV.eGFP and P3HR1-rhLCV.eGFPΔgp350 viruses and incubated for 24 h, followed by DNA extraction and SYBR green-based qPCR using EBV BALF5-, rhLCV EBER- and rhLCV LMP2B-specific primers (Table S4). rhLCV.eGFP BAC plasmid and EBV BALF5 gBlock (Table S4) were amplified as additional rhLCV and EBV positive controls, respectively. Green ticks (☑) and red crosses (☒) represent detectable and undetectable target DNA, respectively. (**C**) Neutralization assay to evaluate EBV complementation during P3HR1-rhLCV.eGFP and P3HR1-rhLCV.eGFPΔgp350 infection. Neutralization experiments were conducted on Raji and BJAB cell lines. Akata-EBV-eGFP (control), P3HR1-rhLCV.eGFP or P3HR1-rhLCV.eGFPΔgp350 were pre-incubated with 25 µg of two EBV neutralizer mAbs: anti-EBV gp350 [72A1] (non rhLCV cross-reactive), and anti-EBV gHgL [AMMO1] (rhLCV cross-reactive). After 1 h incubation the virus/antibody mixtures were used to inoculate Raji and BJAB cells. Viruses pre-incubated with PBS were used as infection positive controls, reaching ~15% infection in both cell lines. Shown is the average % neutralization, defined as the percentual reduction of eGFP-expressing cells in antibody-treated condition vs the respective positive control. All determinations were performed in triplicates.

Table S4: qPCR primers and gBlocks for EBV and rhLCV genomic detection			
**Target**	**Primers**	**Sequence (5'-3')**	**Source**
EBV LMP2B	eLMP2B Fwd	GGCTAATCAGCTTCGAGG	In-house
	eLMP2B Rev	CATCATATTCCATAGTGAGC	
EBV BALF5	eBALF5 Fwd	CGGAAGCCCTCTGGACTTC	(Kimura et al., 1999)
	eBALF5 Rev	CCCTGTTTATCCGATGGAATG	
	gBlock	ATGTCTGGGGGACTCTTCTATAACCCTTTCCTAAGACCTAATA<br>AAGGCCTTCTGAAAAAGCCTGACAAGGAGTACCTGCGTCTCA<br>TTCCCAAGTGTTTCCAGACACCAGGCGCCGCAGGGGTGGTG<br>GATGTGCGGGGGCCTCAGCCCCCCCTGTGCTTCTACCAAGA<br>CTCCCTGACGGTGGTGGGGGGTGACGAGGATGGAAAGGGC<br>ATGTGGTGGCGCCAGCGTGCCCAAGAGGGCACGGCAAGGC<br>CGGAGGCAGACACCCACGGAAGCCCTCTGGACTTCCATGTC<br>TACGACATACTCGAGACGGTGTACACGCACGAGAAATGCGCC<br>GTCATTCCATCGGATAAACAGGGGTATGTGGTGCCATGTGGC<br>ATCGTCATCAAGCTACTGGGCCGGCGCAAGGCCGATGGGGC<br>CAGCGTGTGTGTGAACGTGTTTGGGCAGCAGGCCTACTTCTA<br>CGCCAGCGCGCCTCAGGGTCTGGACGTGGAGTTTGCAGTCC<br>TCAGCGCCCTCAAGGCCAGCACCTTCGACCGCAGGACCCCC<br>TGCCGGGTCTCGGTGGAGAAGGTCACGCGCCGTTCCATTAT<br>GGGCTACGGCAACCATGCCGGC	(Escalante and Reidel et al., 2024)
rhLCV LMP2B	rhLMP2B Fwd	CGTGCCGTTTTGCACGTTCATAAC	In-house
	rhLMP2B Rev	GCCGCAAGTAGCAAGAGTGA	
	gBlock	CCCCTTAACAGGGGGGAGGGGGGGTTGAGGGGCCGGGATG<br>GATGTTGGGGGACTTCGGTTCTTTGCTCTTAAACCTTTGCAA<br>AATTTTTGCGTGCCGTTTTGCACGTTCATAACGTTTTCCTTTG<br>GTTTCCTACAGGACTATGGCGCCTGGATGGCTGCCCGTGGTT<br>TCTGCACCATATCTTTTCTGGCTGGCTGGAATTGCCGCTTCCT<br>GCTTTTCAGCCTCCGTTAGTGCTCTTGTTGTTACTACCGGCCT<br>TGCTCTCTCACTCTTGCTACTTGCGGCCCTGGTTAACTCATAT<br>GCCGCTCAGCGAAGGAAGGCCGTTACTAAGTTAACTATCCTC<br>GTCGGCATTGTTACATGTAAGTATGTCACCCAGACAGAGAACA<br>GTTGTGGAGGCTCGTTGCGCTTGCAACCATACGTTATCCTTG<br>TGTATTCTTTCAGTTTTTGCCATTCTCCTGACATGGATTGTCCA<br>GCCTAAACCGAGAGATGCCATTGTCTTTGCCTTATTGACCGG<br>GGCCGCTGTACTACAGGCCATTTACAGTAAGTAGCAGCGTGC<br>TTGGCTCGGGGGTGTTTACTGAGTAACAGGGTGCTCGACTC<br>AGGGGTGT	In-house
rhLCV EBER	rhEBER32 Fwd	GGAGGAGATGAGTGTGACTTAAATCA	(Rivailler et al., 2004)
	rhEBER148 Rev	TGAACCGAAGAGAGCAGAAACC	
	gBlock	CCCACAGGGATCCAGAAATAGGTGGACATAAAAAGCTAGGGA<br>GTAGAACACCGTTCATACCACCAGATGGCGGGCGTGAGGAG<br>AAAGGAGATATTGTCACCCCGCCCAGTCTCAGTGACGTAATT<br>CGGACCTCAGGGATATTATGACACTGAGCTAGTGAACACCCG<br>GGACCTACGCTGCCCTAGCGGTTATGCTGGGGAGGAGATGA<br>GTGTGACTTAAATCACCCGTCCCGGGTACAAGTCCCGGGTG<br>GTGAAGACGGGGTCTGGTGCTGCTAAAAGTTTGGACAGTCC<br>CCGGTTTCTGCTCTCTTCGGTTCAAACCAGCTGGTGGTCCGC<br>CTGTTTTGTTGCTGCTTTTGCTTTCAGTTTTGAAACCCTGTTA<br>TTATAGCGCTTGCACCTGGTTCTCCCGGTGCTATTTTTGGCCA<br>ACAAGGCCGCCGTGACGTAGCATAACCTTGAGTTCTTTGATG<br>TTCAAAACCAGTGAAGCAGTTAGGACATTGGCTAGCCTAGAG<br>GTTACGGACAGAGGGGGGTGGTGTGCGGGTTCATCCGTCCG<br>TGCCGAGTACAAGTCCCGGGAAGGGGAGAAGAGCGGCTCC<br>CGCCTATGTGCAATTGA	In-house

A **Expression levels of eGFP cassette-adjacent genes in induced rhLCV producer cell lines**

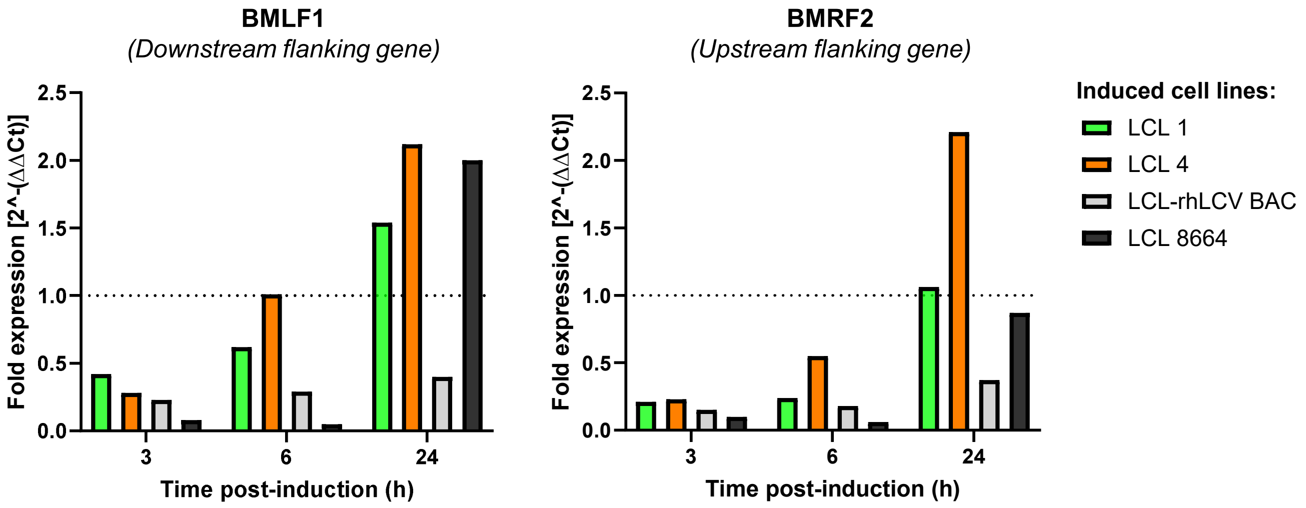

B **qPCR primers used for rhLCV gene expression detection**

Target	Primers	Sequence (5'-3')	Amplicon size (bp)	Source
rhBMLF1	rhBMLF1 Fwd	TCAATGTGCCGGGGTTGTAA	89	In-house
	rhBMLF1 Rev	GGCCTACATCAACGCTCACT		
rhBMRF2	rhBMRF2 Fwd	GCTGTCTTCATGTCCCCCTT	116	In-house
	rhBMRF2 Rev	CAGAAAATGCTGCGTCTCCG		
RPL13 A	rmRPL13A Fwd (Exon 7)	CCTGGAGGAGAAGAGGAAAGAGA	126	(Ahn et al., 2008)
	rmRPL13A Rev (Exon 8)	TTGAGGACCTCTGTGTATTTGTCAA		

Figure S7. Expression assessment of the eGFP cassette-immediate adjacent genes BMLF1 and BMRF2. (**A**) Relative expression levels of BMLF1 and BMRF2 in the indicated induced rhLCV LCLs. Induced and uninduced LCLs were harvested at 3, 6, and 24 h post-induction for RNA extraction, followed by reverse transcription and SYBR-based qPCR. Gene expression was normalized to the housekeeping gene, rhesus macaque RPL13A gene, and reported according to the ΔΔCT method, as the relative expression to the uninduced control samples. Shown is the mean of two technical replicates. (**B**) qPCR primers used to study the expression levels of BMLF1 and BMRF2.

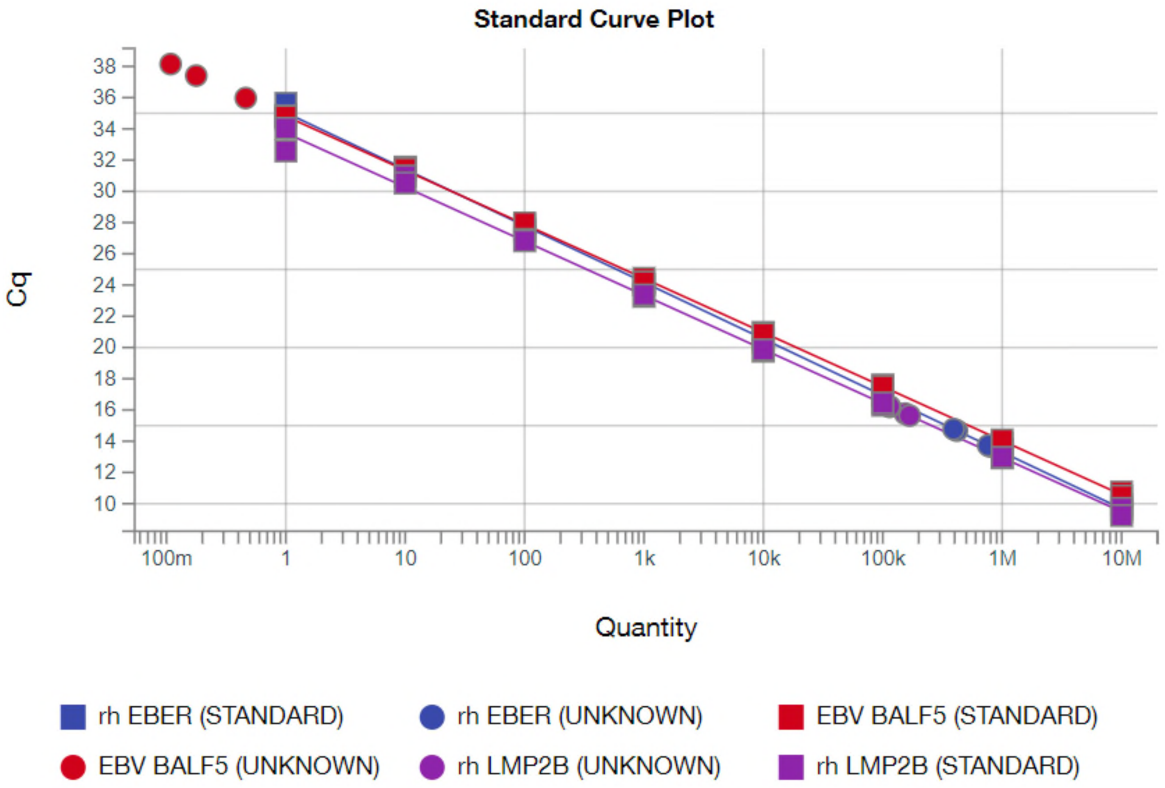

Target: rh EBER Slope: -3.622 R^2: 0.999 Y-Inter: 35.038 Eff%: 88.824 Error: 0.027

Target: EBV BALF5 Slope: -3.463 R^2: 1 Y-Inter: 34.809 Eff%: 94.444 Error: 0.013

Target: rh LMP2B Slope: -3.464 R^2: 0.998 Y-Inter: 33.733 Eff%: 94.406 Error: 0.043

Figure S8. rhLCV LMP 2B, rhLCV EBER, and EBV BALF5 standard curves for rhLCV and EBV genomic quantification. rhLCV LMP 2B, rhLCV EBER, and EBV BALF5 standard curves generated with gBlocks of the corresponding target genes. Standard curves were constructed by a 1:10 serial dilution (10^7 to 1 copy/µl) of gBlocks comprising DNA fragments of rhLCV EBER, rhLCV LMP2B, and EBV BALF5 for their respective genomic viral DNA quantification. Shown are representative standard curve plots obtained using the QuantStudio Design and Analysis Software, Thermo Fisher Scientific.

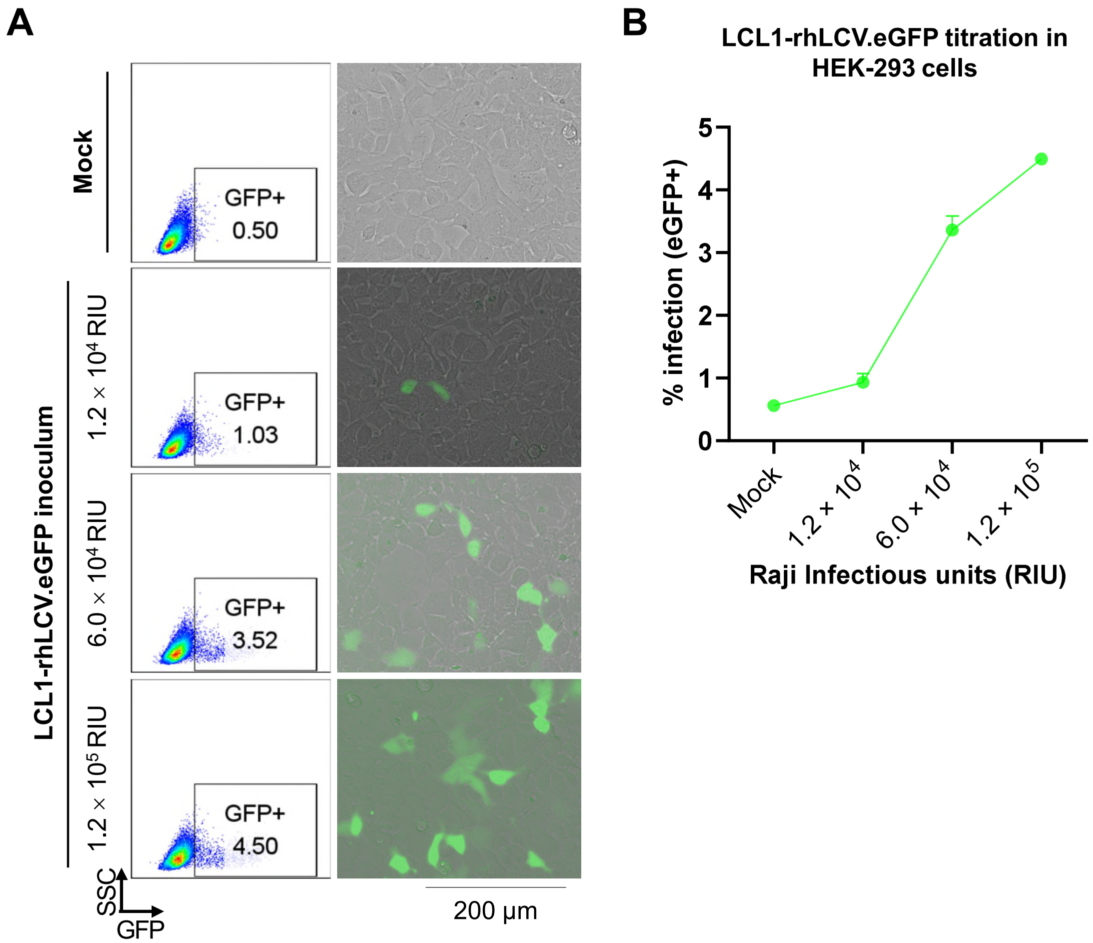
A
Mock
LCL1-rhLCV.eGFP inoculum
1.2 × 10^4 RIU
6.0 × 10^4 RIU
1.2 × 10^5 RIU
GFP+
0.50
GFP+
1.03
GFP+
3.52
GFP+
4.50
SSC
GFP
200 μm
B
LCL1-rhLCV.eGFP titration in HEK-293 cells
% infection (eGFP+)
0
1
2
3
4
5
Mock
1.2 × 10^4
6.0 × 10^4
1.2 × 10^5
Raji Infectious units (RIU)

Figure S9. LCL1-rhLCV.eGFP titration on HEK-293 cells. (**A**) Microscopy analysis of HEK-293 cells infected with different RIU of purified LCL1-rhLCV.eGFP. Based on the previously obtained LCL1-rhLCV.eGFP RIU/mL, HEK-293 cells were infected with the listed doses of LCL1-rhLCV.eGFP and incubated for 24 h. Shown are representative side scatter (SSC) vs eGFP flow cytometry plots (left) and phase-GFP-channel-merged micrographs (right) of triplicate infected samples. (**B**) Flow cytometry analysis of HEK-293 cells infected with different volumes of purified LCL1-rhLCV.eGFP virus. Cells from (A) were harvested and processed for flow cytometry analysis of eGFP expression. Shown is the mean + SEM quantification of infected (eGFP-expressing) cells at each volume for triplicates, which was used to select the RIU needed to achieve an ~3% infection in HEK-293 cells in following experiments.
